# Comprehensive analysis of silk proteins and gland compartments in *Limnephilus lunatus*, a case-making trichopteran

**DOI:** 10.1186/s12864-024-10381-4

**Published:** 2024-05-14

**Authors:** Lenka Rouhova, Martina Zurovcova, Miluse Hradilova, Michal Sery, Hana Sehadova, Michal Zurovec

**Affiliations:** 1 Biology Centre of the Czech Academy of Sciences, Institute of Entomology, Ceske Budejovice, Czech Republic; 2 Faculty of Science, University of South Bohemia, Ceske Budejovice, Czech Republic; 3 Institute of Molecular Genetics, Academy of Sciences of the Czech Republic, Prague, Czech Republic

**Keywords:** Fibroin, Sericin, Hydrophobicity, Gene duplication, *Limnephilus flavicornis*, *Plectrocnemia conspersa*

## Abstract

Caddisfly larvae produce silk containing heavy and light fibroins, similar to the silk of Lepidoptera, for the construction of underwater structures. We analyzed the silk of *Limnephilus lunatus* belonging to the case-forming suborder Integripalpia. We analyzed the transcriptome, mapped the transcripts to a reference genome and identified over 80 proteins using proteomic methods, and checked the specificity of their expression. For comparison, we also analyzed the transcriptome and silk proteome of *Limnephilus flavicornis.* Our results show that fibroins and adhesives are produced together in the middle and posterior parts of the silk glands, while the anterior part produces enzymes and an unknown protein AT24. The number of silk proteins of *L. lunatus* far exceeds that of the web-spinning *Plectrocnemia conspersa*, a previously described species from the suborder Annulipalpia. Our results support the idea of increasing the structural complexity of silk in rigid case builders compared to trap web builders.

**Supplementary Information:**

The online version contains supplementary material available at 10.1186/s12864-024-10381-4.

## Introduction

Silk is a protein-based material produced by many arthropod species and is characterized by remarkable mechanical properties. The silk of moths and their sister group, the caddisflies, is produced by transformed salivary glands, the silk glands (SGs). The silks in both groups contain a fibrous core of proteins called fibroin heavy and light chains (FibH and FibL). The core formed by the axial filament is coated by adhesive proteins and small amounts of additional proteins of poorly known function [1, 2]. FibH and FibL are produced in the posterior part of the SGs in *B. mori* and other moths, while sericins and other additives are secreted from the middle SG region [3]. The localization of the production of individual silk components in parts of the SG has not been studied in detail in Trichoptera, except for *P. conspersa*.

The architecture of the silk structures formed by caddisfly larvae is closely related to their function and the phylogenetic position of the species [4]. The structure of the silk secretion of case-forming caddisfly species belonging to the suborder Integripalpia is characterized by ribbon-like fibers attached to the surface of the object to which they adhere [5]. The connection of the two threads is visible in the form of a seam in the middle of the ribbon. This is typical of species that use silk to bond different natural materials in the construction of their shells, including *Hesperophylax consimilis* [5, 6], *Hesperophylax occidentalis* [7, 8], *Neophylax concinnus* [9] *Hydatophylax nigrovittatus* [10], *Drusus improvisus* [11]*,* and *Limnephilus vittalus* [11]. In contrast, the SG of predatory species such as *Plectrocnemia conspersa* [1]*, Hydropsyche pellucidula* [11] and *Parapsyche elsis* [12] (suborder Annulipalpia) produce more distinct fibers to build self-supporting capture nets and retreats.

Thus far, very little is known about the composition of caddisfly silk. With few exceptions, only the sequences of heavy and light fibroin (FibH, FibL) have been described for a few species, as parts of their molecules are relatively conserved, except for the central repetitive FibH region [12, 13]. A more detailed study of the silk of *Hesperophylax occidentalis* from the family Limnephilidae identified several silk proteins in addition to fibroins, including a putative structural protein called PEVK-like (PEVK) and two enzymes that may be involved in cross-linking silk proteins, namely peroxidase of the peroxinectin (Pxt) subfamily and superoxide dismutase 3 (SOD3) [8].

The most comprehensive previous analysis of silk has been performed on *P. conspersa*, a caddisfly of the suborder Annulipalpia [1]. Its larvae are predatory and build underwater nets to catch their prey. A detailed analysis of their silk revealed 27 major silk protein candidates, including FibH, FibL zonadhesin-like proteins (Zon), mucins (Muc), and several components with unknown functions that have no obvious homology to proteins in moth silks [1]. Furthermore, no clear homologs of moth sericins have been identified, and it can be assumed that the adhesive proteins of caddisflies are rather different from their soluble counterparts in moths. It is likely that the composition of silk also differs between distantly related trichopteran groups, with the case-making limnephilids (belonging to the suborder Integripalpia) relying more on their adhesion to natural materials (small stones, sticks, or leaf fragments) than the strong fibers of predatory species (such as *P. conspersa*) that produce self-supporting silk webs.

We have identified over eighty candidate genes in *L. lunatus* that are responsible for the synthesis of the SG secretory products, demonstrated the tissue-specificity of their expression and provided information on their exon–intron structure and chromosomal localization. Our research provides valuable insights into the molecular aspects of silk production in case-making trichopterans and reveals the diversity of silk composition in different caddisfly species. The identification of specific proteins provides a basis for further understanding their functions, as well as the ecological and evolutionary adaptations associated with silk production in these organisms.

## Results

### The morphology of silk glands and silk fibers

**Fig. 1:**
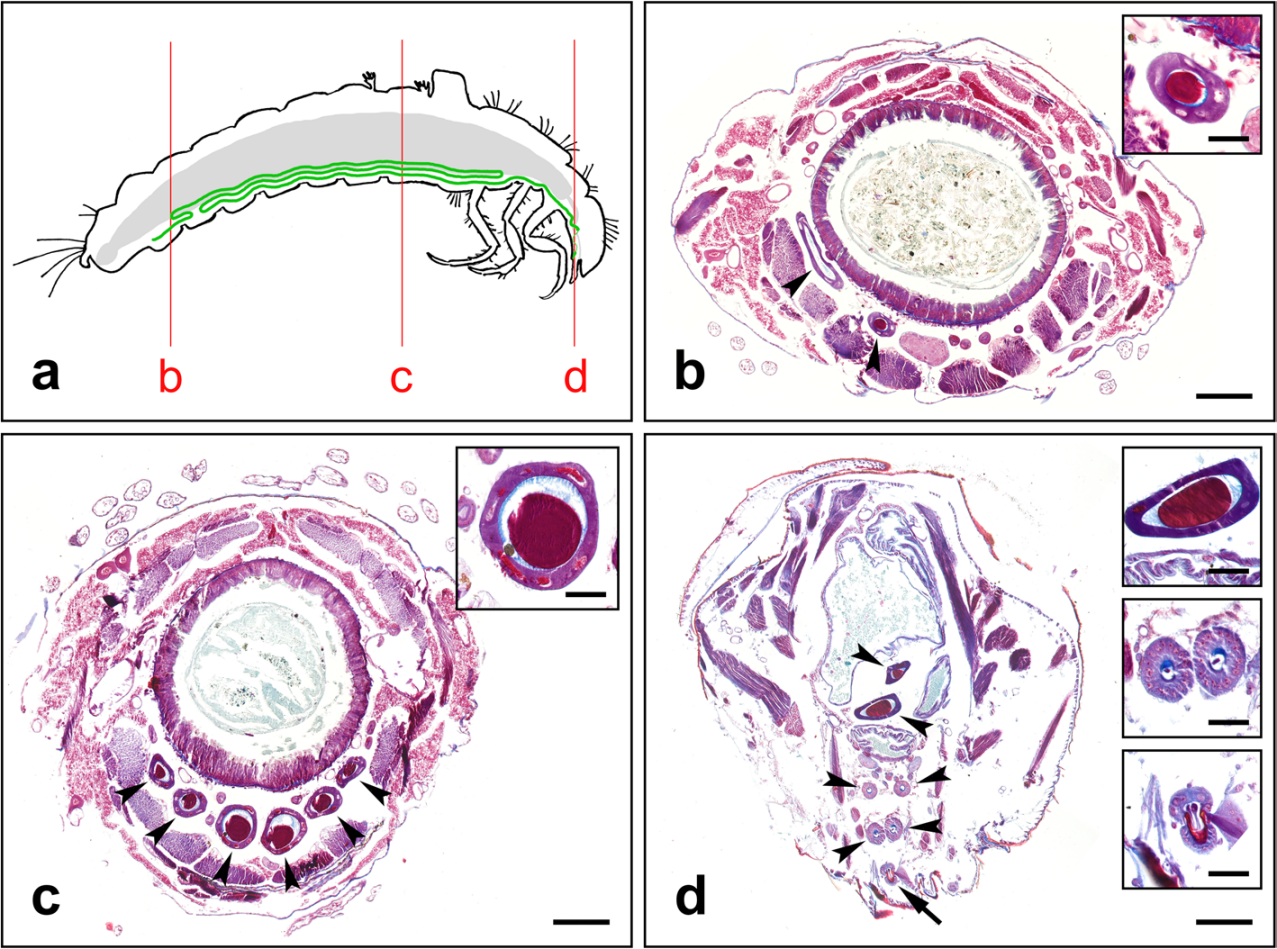
Morphology of SGs in *L. lunatus* larva. **a** Schematic drawing representing the position of the SGs (in green) in *L. lunatus* larva. The intestine (in gray) is depicted for visual reference. Vertical red lines show the position of the transverse whole-body sections (b-d). **b** The rearmost part of the SG. **c** The middle part of the SG where it is pleated into three parallel folds. **d** The part of the head where the MSGs narrow down into ASGs and enter the spinneret. Sections through the SGs are marked by arrowheads; the arrow in **d** marks the spinneret. Enlarged inset images show representative sections of the silk glands. Scale bars: b-d 200 µm; inset images 50 µm

The SGs of L. *lunatus* are located on the ventral side of the larval body. For most of their length, the SGs are arranged in a Z-shaped fold that extends from the metanotum to about the seventh abdominal segment. The SGs are relatively thin and long and reach at least twice the length of the larval body when stretched (Fig. 1a). The three main folds of a SG are arranged so that the most posterior fold is on the top (Fig. 1). The anterior SG (ASG), which is located in the head region, is shorter and thinner than the remainder of the SGs. The rear SGs include the posterior and the middle parts (PSG and MSG) of the SGs, which are not clearly separated morphologically. A considerable part of the volume of the SG is constituted of the liquid silk stored in the lumen of the gland. The rear SGs thicken toward the ASG because the amount of secretory material in the lumen increases. The secretory material consists of two layers – an axial filament that is stained red with the Masson trichome staining method and a relatively thin coating layer that is stained blue (Fig. 1). It appears that the SG cells of *L. lunatus* produce both the fibroin core and the envelope in the same compartment and yet these proteins form separate layers.

**Fig. 2:**
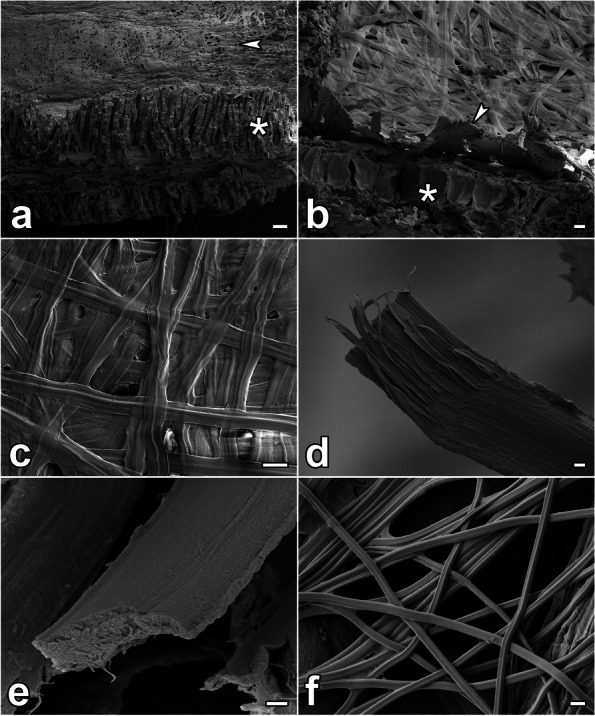
SEM photographs of the silk of *L. lunatus* and *P. conspersa*. **a**-**b** view of a cross-section (fracture) of the protective case, which is strengthened with pieces of plant material. Its inner side is covered by silk fibers (arrowhead). The asterisk labels remnants of the leaf that was used as building material. **c** Detailed view of the inner surface of the case. The fibers are flat, and they pliantly adhere to the substrate. **d**-**e** Fractures of the *L. lunatus* fibers. The torn fibers tend to split into longitudinal filaments. The coating layer of adhesives is quite thin. **f** For comparison, *P. conspersa* silk shows different morphology. Scalebars: **a** 100 µm; **b**-**c** 10 µm; (d-f) 1 µm

As depicted in Fig. 2, the silk from both SGs fuses into a single flat ribbon that is approximately 8 µm wide. The break in the fiber reveals the longitudinal arrangement of the fibrils (Fig. 2d). Interestingly, the adhesive layer of the coating is not clearly visible, thereby indicating that it forms only a thin film on the fiber surface. The fibers of *L. lunatus* adhere to small fragments of vegetation and take on their shape, which is in contrast to the clearly defined shape of the fibers of *P. conspersa* (Fig. 2f)*.* Obviously, the silk of *L. lunatus* is more malleable during spinning as compared to that of *P. conspersa*, which is probably due to the different use of silk between these two species.

### Identification of the genes encoding silk proteins

To comprehensively analyze the genes involved in silk synthesis, we isolated RNA from the silk glands of *L. lunatus* and generated the SG-specific transcriptome. Eighteen million paired-end reads were assembled de novo using Trinity software. The assembly of the transcriptome yielded 57,519 contigs with a length of 0.2 kb–10 kb, and the complete BUSCO gene coverage was 71.6%. To identify the silk-specific transcripts, we performed a proteomic analysis of the spun silk of *L. lunatus* using tryptic peptide mapping. As presented in Table S1, we identified 127 proteins (395 peptides), of which 58 contained a signal peptide for secretion. A few proteins were identified by only a single peptide. The sequences of the candidate proteins and their transcripts were manually curated based on the available genome sequence. This also led to the discovery of putative additional paralogs of the discovered proteins that are encoded by neighboring clustered genes and might have been overlooked in the previous proteomic analysis.

**Fig. 3:**
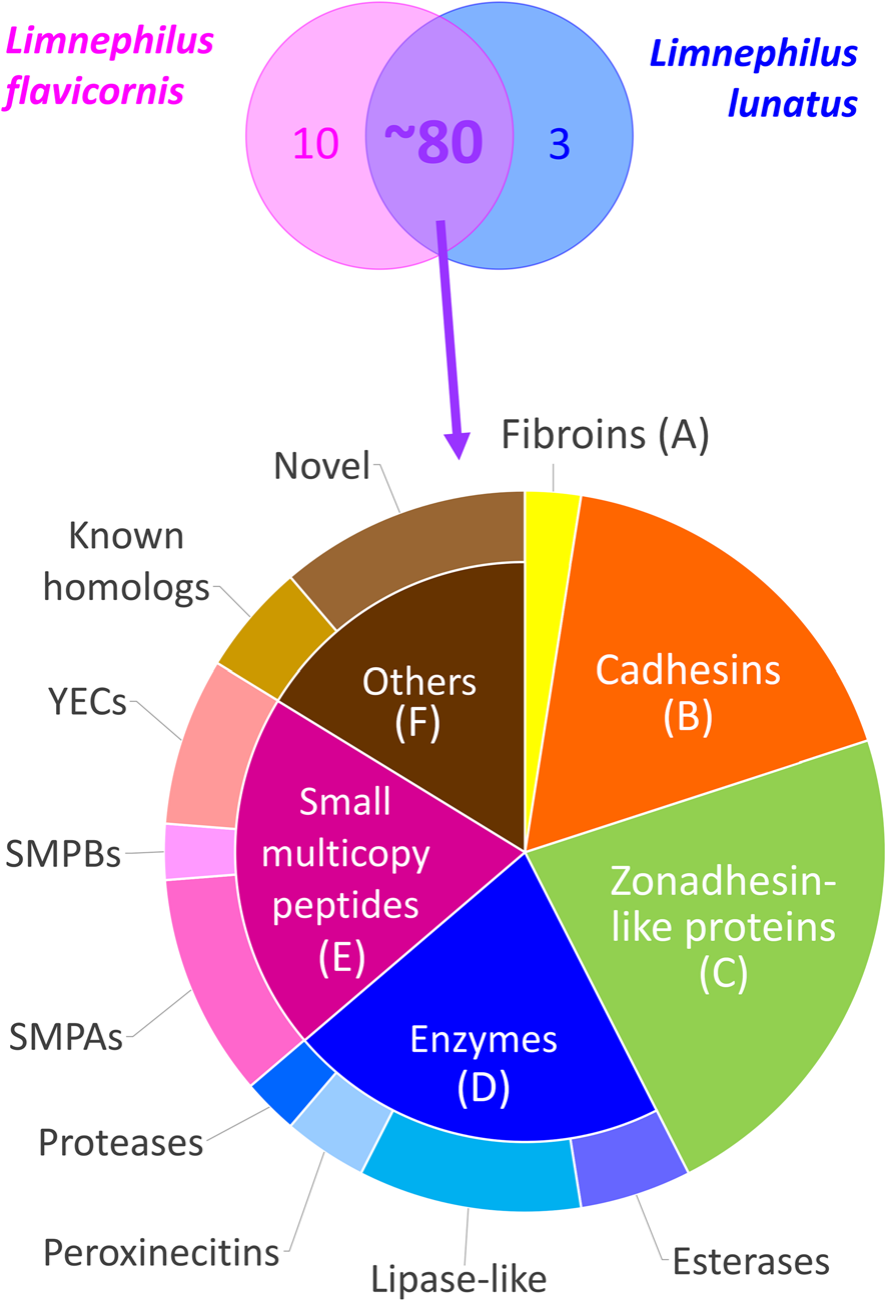
Comparison of the silk proteomes of *L. lunatus* and *L. flavicornis* and categorization of the resulting candidates into six classes. The list of overlapping proteins was expanded by analyzing the genome of *L. lunatus* to include paralogs and other members of gene families that were not detected during MS analysis

To validate the proteins identified by a single peptide and complement our candidate list with unidentified proteins, we constructed a transcriptome and performed an analysis of secretory proteins from a closely related species, *L. flavicornis*. The assembly contained 49,463 transcripts with 69% complete BUSCOs. The proteome of *L. flavicornis* contains 633 peptides that belong to 115 proteins (Table S2); 75 of these 115 have a signal peptide. A comparison and a complementary search in the databases of both species revealed that both silks have a rather similar protein composition. However, a few proteins occurred in unequal numbers of paralogs between the two species. For example, there is a duplication of cadhesin 6 (Caz6) and zonadhesin-like proteins 1 and 2 (Zon1, Zon2) in *L. flavicornis*. In addition, the number of silk proteins believed to be common between *L. lunatus* and *L. flavicornis* was expanded by identifying putative candidates when analyzing the *L. lunatus* genome. The final number of proteins amounts to approximately 80. In contrast, the number of identified secreted proteins specific to only one species was rather low: 3 in *L. lunatus* and 10 in *L. flavicornis* (Fig. 3). This confirms that most of the proteins identified in the silk of *L. lunatus* correspond to those of *L. flavicornis* and that the analyses were sufficiently detailed/robust and led to similar results. This reduces the possibility that their presence in the silk is coincidental.

Table 1 provides an overview of the silk protein candidates of *L. lunatus*. It includes information on whether they were identified in the proteome or inferred from the genome, their predicted size (excluding signal peptide) and the most abundant amino acids. As indicated in Table 1, there are a few patterns in amino acid content that characterize some of the protein groups. Zons, for example, are rich in Cys, which typically accounts for 12%–17% of all amino acid residues. In comparison, Caz typically contain a high proportion of Ser or Thr, which can reach over 40% of the residues (Table 2). This makes them similar to the sericins of Lepidoptera, although there is no sufficiently conserved pattern in their sequence to prove their homology. Another amino acid characteristic of several silk components is Tyr, which accounts for over 20% in small multicopy peptides A (SMPAs) and at least 10% in small YEC-rich multicopy peptides (YECs) (Table 1).

**Table 1:** The list of silk protein candidates in *L. lunatus*. It includes the full names, the GenBank identifiers, protein category (A – fibroins; B– cadhesins; C – zonadhesin-like proteins; D – enzymes; E – small multicopy peptides, F – others), information on the detection method (Det), the protein size, and the three most abundant amino acids (each measured without the signal peptide). Abbreviations: P – protein was detected in the silk by proteomics; G – related proteins added based on genomic analysis

Symbol	Full name	GenBank	Cat	Det	Size [kDa]	1st AA (%)	2nd AA (%)	3rd AA (%)
FibH	Fibroin heavy chain	BK063451	A	P	997	G (27.8)	S (17.2)	R (14.7)
FibL	Fibroin light chain	BK062791	A	P	24	A (12.7)	L (11.8)	S (10.5)
Caz1	Cadhesin 1	BK062803	B	P	34	T (32.7)	C,K (15.9)	P (10.0)
Caz2A	Cadhesin 2A	BK062803	B	G	68	S (22.7)	A (17.0)	T (8.8)
Caz2B	Cadhesin 2B	BK062803	B	G	27	S (25.2)	A (17.2)	T (8.8)
Caz3A	Cadhesin 3A	BK062803	B	P	40	S (37.5)	A (21.7)	N (13.6)
Caz3B	Cadhesin 3B	BK062803	B	P	56	S (27.7)	A (22.6)	N (18.2)
TSAG18	TSAG-rich 18 kDa protein	BK062803	B	P	18	T (17.0)	S (14.3)	A (13.7)
Caz4	Cadhesin 4	BK062803	B	P	84	T (40.1)	A (22.3)	P (17.4)
Caz5	Cadhesin 5	BK062803	B	G	48	T (45.0)	P (12.9)	K (11.8)
Caz6	Cadhesin 6	BK062803	B	P	36	T (13.8)	S (11.9)	A (11.6)
Caz7	Cadhesin 7	BK062803	B	G	121	S (40.4)	A (21.3)	T (13.4)
Caz8	Cadhesin 8	BK062803	B	G	89	S (46.5)	A (23.4)	T (12.0)
Caz9	Cadhesin 9	BK062803	B	P	95	S (47.2)	A (26.7)	K (8.7)
Caz10	Cadhesin 10	BK062804	B	P	222	S (19.4)	P (10.4)	A (9.0)
Caz11	Cadhesin 11	BK062804	B	P	125	S (24.5)	T (15.0)	A (10.5)
Caz12	Cadhesin 12	BK062804	B	P	32	S (19.5)	G (15.2)	A (11.1)
Zon1	Zonadhesin-like protein 1	BK062784	C	P	105	C (16.4)	K (9.7)	P (9.1)
Zon2	Zonadhesin-like protein 2	BK062787	C	P	49	C (14.8)	P (12.4)	K (10.3)
Zon3A	Zonadhesin-like protein 3A	BK062786	C	P	16	C (12.2)	P,S (9.5)	R,G (8.8)
Zon3B	Zonadhesin-like protein 3B	BK062809	C	P	17	C (11.8)	R,G (11.2)	P (8.6)
Zon4	Zonadhesin-like protein 4	BK062787	C	P	73	T (14.8)	C (14.1)	P (10.9)
Zon5	Zonadhesin-like protein 5	BK062787	C	G	38	C (13.2)	P (9.8)	K (8.7)
Zon6	Zonadhesin-like protein 6	BK062793	C	P	27	C (13.4)	P (9.1)	A,N (8.3)
Zon7	Zonadhesin-like protein 7	BK062793	C	G	33	C (13.8)	K (9.7)	E (7.6)
Zon8	Zonadhesin-like protein 8	BK062812	C	P	54	C (15.3)	P (13.1)	K (8.0)
Zon9	Zonadhesin-like protein 9	BK062805	C	P	48	C (14.6)	K (9.8)	P,T (9.6)
Zon10	Zonadhesin-like protein 10	BK062805	C	P	58	C (14.7)	T (11.8)	K,P (9.5)
Zon11	Zonadhesin-like protein 11	BK062808	C	P	53	C (14.5)	P (8.3)	K (7.9)
Zon12A	Zonadhesin-like protein 12A	BK062787	C	P	27	C (17.3)	E (10.7)	G (7.4)
Zon12B	Zonadhesin-like protein 12B	BK062787	C	P	40	C (15.8)	K (9.0)	P (8.5)
Zon13A	Zonadhesin-like protein 13A	BK062785	C	P	53	C (14.0)	P (10.1)	K (8.7)
Zon14	Zonadhesin-like protein 14	BK062815	C	G	34	C (15.1)	P (14.5)	G (11.6)
Zon15	Zonadhesin-like protein 15	BK062814	C	G	30	C (15.5)	P (10.0)	E (9.6)
PDCPI	Pacifastin domain-containing prot. inh	BK062805	C	G	25	T (23.4)	C (13.2)	K (9.8)
Est1	Esterase 1	BK062795	D	P	59	L (9.2)	V (8.3)	P (7.7)
Est2	Esterase 2	BK062795	D	P	59	L (9.2)	G (7.9)	V (7.7)
Est3	Esterase 3	BK062795	D	P	60	L (9.2)	P (7.8)	G (7.6)
Est4	Esterase 4	BK062796	D	G	60	L (9.7)	G (8.4)	S (7.4)
PlipA1	Pancreatic lipase-related protein A1	BK062789	D	G	35	T (10.3)	S (9.7)	L (9.4)
PlipA2	Pancreatic lipase-related protein A2	BK062789	D	G	36	G,T (9.7)	L,S (8.2)	A (7.6)
PlipA3	Pancreatic lipase-related protein A3	BK062789	D	P	35	T (11.0)	G (10.4)	L (10.1)
PlipA4	Pancreatic lipase-related protein A4	BK062789	D	G	36	T (10.7)	L (9.5)	S (8.6)
PlipB	Pancreatic lipase-related protein B	BK062789	D	G	36	G (9.8)	I,S,T (8.9)	L (8.0)
PlipC	Pancreatic lipase-related protein C	BK062788	D	P	39	G,L (9.4)	T (8.6)	S (8.3)
PlipD1	Pancreatic lipase-related protein D1	BK062790	D	G	34	L (12.6)	T (9.5)	G (9.1)
PlipD2	Pancreatic lipase-related protein D2	BK062790	D	G	35	L (12.9)	G (9.5)	N,S,T (7.9)
Pxn1_X1	Peroxinectin 1—transcr. variant X1	BK062798	D	P	82	P (8.2)	A (8.1)	S (7.3)
Pxn1_X2	Peroxinectin 1—transcr. variant X2	BK062798	D	P	74	P (7.6)	L (7.3)	T (7.1)
Pxn2	Peroxinectin 2	BK062798	D	P	82	P (7.2)	A (6.7)	N,G,T (6.6)
Pxn3	Peroxinectin 3	BK062799	D	G	71	L (8.0)	N (7.5)	K (6.7)
SP1	Serine protease 1	BK062802	D	G	41	G,T (9.3)	S (8.0)	V (7.4)
SP2	Serine protease 2	BK062802	D	P	41	T (12.3)	G (10.2)	L (7.9)
YEC1	Small YEC-rich multicopy peptide 1	BK062783	E	G	6	Y (13.0)	C (11.1)	P (9.3)
YEC2	Small YEC-rich multicopy peptide 2	BK062782	E	G	7	Y (13.3)	C,V (10.0)	D (8.3)
YEC3	Small YEC-rich multicopy peptide 3	BK062782	E	P	7	Y,E (12.1)	C (10.3)	A (8.6)
YEC4	Small YEC-rich multicopy peptide 4	BK062782	E	P	7	Y,E (11.7)	C,A (10.0)	R,T (8.3)
YEC5	Small YEC-rich multicopy peptide 5	BK062782	E	G	8	Y (13.4)	E (10.4)	D,C (9.0)
YEC6	Small YEC-rich multicopy peptide 6	BK062782	E	G	7	E (13.3)	Y,C (10.0)	D (8.3)
SMPA1	Small multicopy peptide A1	BK062800	E	P	4	Y (20.7)	D,P (13.8)	R (10.3)
SMPA2	Small multicopy peptide A2	BK062800	E	P	4	Y (20.7)	D,P (13.8)	R (10.3)
SMPA3	Small multicopy peptide A3	BK062800	E	G	4	Y (28.1)	D (18.8)	N,K (15.6)
SMPA4	Small multicopy peptide A4	BK062800	E	P	4	Y (28.1)	K (18.8)	N,D (15.6)
SMPA5	Small multicopy peptide A5	BK062800	E	P	4	Y (27.6)	K (20.7)	N,D (13.8)
SMPA6	Small multicopy peptide A6	BK062800	E	P	4	Y (28.1)	K (18.8)	N,D (15.6)
SMPA7	Small multicopy peptide A7	BK062800	E	P	3	Y (29.6)	K (22.2)	N (14.8)
SMPA8	Small multicopy peptide A8	BK062800	E	P	3	Y (29.6)	K (22.2)	N (14.8)
SMPB1	Small multicopy peptide B1	BK062800	E	P	4	G (18.4)	K (15.8)	L,P (10.5)
SMPB2	Small multicopy peptide B2	BK062800	E	P	8	G (17.1)	H (15.7)	S (12.9)
KD15	KD-rich 15 kDa protein	BK062801	F	P	15	K (14.8)	D (9.6)	G,L (8.1)
PEVK	PEVK-like protein	BK062794	F	P	57	E (19.6)	A (19.2)	V (14.8)
LAN32	LAN32 homolog	BK062792	F	P	31	L (10.1)	T (9.0)	S (8.3)
LA27	LA rich 27 kDa protein	BK062792	F	P	27	L (11.1)	A (8.3)	K,S (7.9)
LS29	LS rich 29 kDa protein	BK062792	F	P	29	L (11.3)	S (9.1)	A,E (7.9)
AT24A	AT rich 24 kDa protein A	BK062780	F	G	24	A (18.6)	T (10.0)	I,V (9.1)
AT24B	AT rich 24 kDa protein B	BK062780	F	P	24	A (16.9)	T (11.3)	I,V (9.1)
C30A	C-rich 30 kDa protein A	BK062797	F	P	30	C (11.7)	K (9.01)	G (8.3)
C30B	C-rich 30 kDa protein B	BK062797	F	P	29	C (11.6)	G (9.4)	I (8.2)
C30C	C-rich 30 kDa protein C	BK062797	F	G	31	C (11.0)	E (10.7)	K (10.3)
UchC1	Unchar. conserved CG3556-like 1	BK062781	F	P	15	T (9.9)	S,V (8.4)	P (7.6)
UchC2	Unchar. conserved CG3556-like 2	BK062781	F	G	14	I (10.4)	K,V (8.0)	S,T (7.2)

**Table 2:** Similarity of *L. lunatus* silk proteins with those found in other species of caddisflies. Fibroins and zonadhesin-like proteins are not included because of the high number of known homologs and difficulty to determine orthologs, respectively

Protein	Known silk homolog	Organism	Protein identity/ similarity (%)	Reference
L.lun-PEVK	PEVK-like (KM384739)	*Hesperophylax occidentalis*	87.1/90.1	Wang et al. 2014 [8]
L.lun-Pxn1	Pxt (KM384736)	*Hesperophylax occidentalis*	89.1/92.6	Wang et al. 2014 [8]
	S.ang100.134	*Stenopsyche angustata*	19.2/26,0	Wang et al. 2023 [2]
L.lun-Pxn2	Pxt (KM384736)	*Hesperophylax occidentalis*	73.3/79.7	Wang et al. 2014 [8]
	S.ang100.134	*Stenopsyche angustata*	18.1/24.0	Wang et al. 2023 [2]
L.lun-Pxn3	Pxt (KM384736)	*Hesperophylax occidentalis*	74.5/82.5	Wang et al. 2014 [8]
L.lun-LAN32	LAN32 (OL791320)	*Plectrocnemia conspersa*	20.4/35.6	Rouhova et al. 2022 [1]
	S.ang97.245	*Stenopsyche angustata*	22.1/42.3	Wang et al. 2023 [2]
L.lun-Caz12	SGA28 (OL589401)	*Plectrocnemia conspersa*	29.5/38.0	Rouhova et al. 2022 [1]
	S.ang7.801.2	*Stenopsyche angustata*	13.6/17.8	Wang et al. 2023 [2]
L.lun-SP2	S.ang157.465	*Stenopsyche angustata*	57.7/71.2	Wang et al. 2023 [2]
Lun-Est1	S.ang133.18	*Stenopsyche angustata*	37.1/54.6	Wang et al. 2023 [2]
Lun-Est2	S.ang133.18	*Stenopsyche angustata*	36.7/54.4	Wang et al. 2023 [2]
Lun-Est3	S.ang133.18	*Stenopsyche angustata*	36.0/55.9	Wang et al. 2023 [2]

### Tissue specificity in the expression of candidate silk genes

**Fig. 4:**
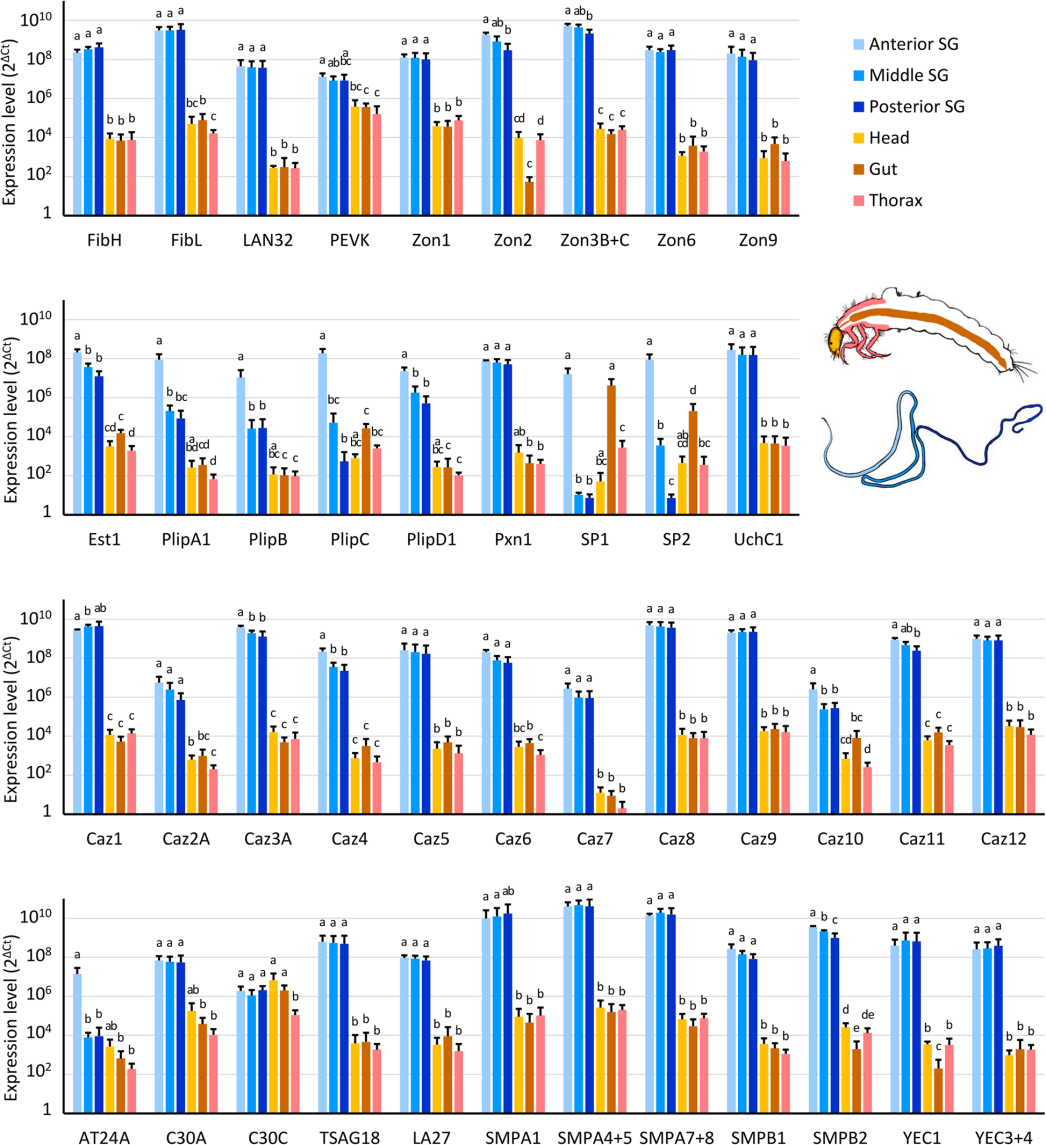
Tissue specificity of expression of candidate silk genes. Quantitative PCR (qRT-PCR) was used to assess the expression of 42 representative transcripts derived from silk candidate genes in six tissue types. The scale used is logarithmic, and differences in statistical significance determined by the Kruskal–Wallis rank sum test and pairwise Wilcoxon test are indicated by different letters. Samples with the same letter indicate that there is no significant difference between them

To confirm the expression specificity of candidate silk genes in SGs, we analyzed the transcription of representative candidate genes by quantitative real-time PCR (qRT-PCR) using RNA samples from different tissues, including intestine, head, and thorax and three approximately equal-sized parts of SG separated at glandular folds. As shown in Fig. 4, the expression of 41 of the 42 genes analyzed was specific for SG (*p* < 0.05). The only exception is serine protease 1 (SP1), which is also highly expressed in the gut. In contrast to the Lepidoptera, the expression of genes encoding the fibroin core and most other silk candidate genes is rather uniform in the rear SG. For example, transcription of *fibH* and *fibL* occurred in all parts of the SG. This is consistent with the morphological observations that the rear SG is not divided into MSG and PSG. In contrast, most enzymes (with the exception of Pxn1) and a novel gene encoding an AT-rich 24 kDa protein A (AT24A) were preferentially expressed in the part of the SG that included the ASG, thereby suggesting that the ASG may represent a separate compartment that secretes different proteins than the rear SG (Fig. 4).

### Structure and chromosomal localization of candidate silk genes

**Fig. 5:**
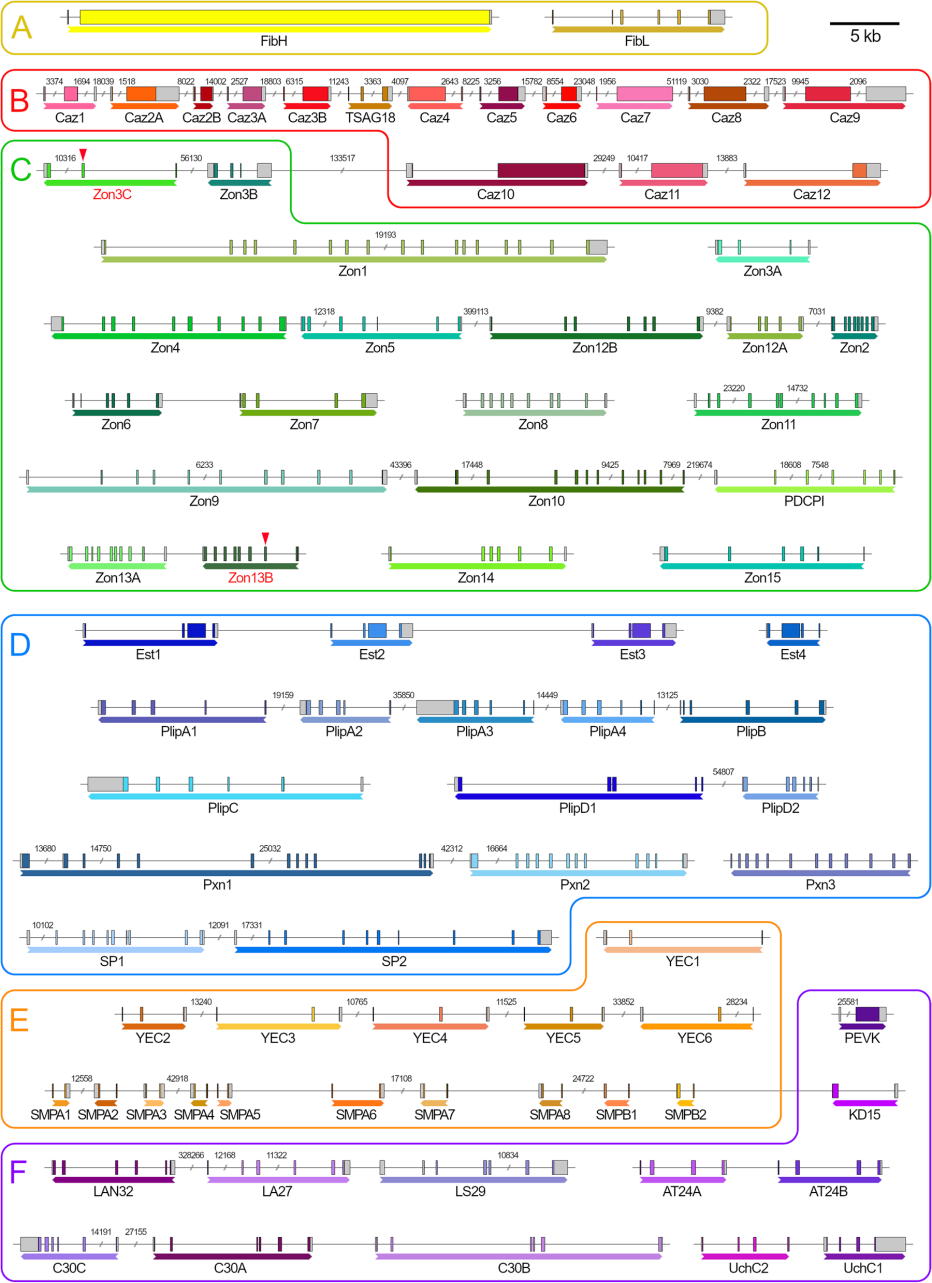
Genomic structure of 82 silk candidates. The genes encoding putative silk proteins and peptides can be divided into six classes, as depicted in Fig. 3. Untranslated regions (UTRs) are depicted in gray, and coding regions are depicted in color. Genes for Fibroins (group **A**) are shown in yellow, *caz* genes are depicted in shades of red (group **B**), *Zon* genes (group **C**) are depicted in green, while genes for enzymes (group **D**) are depicted in blue. Further, the genes of the *SMP* cluster (group **E**) are marked in orange, and the remaining genes collected in group F are depicted in purple. The arrows below each gene indicate the 5’-3’ direction. The length scale is located in the upper right corner, and the scale breaks in intergenic regions or introns are labeled with the actual lengths of the respective regions. For full names and accession numbers, see Table 2

**Fig. 6:**
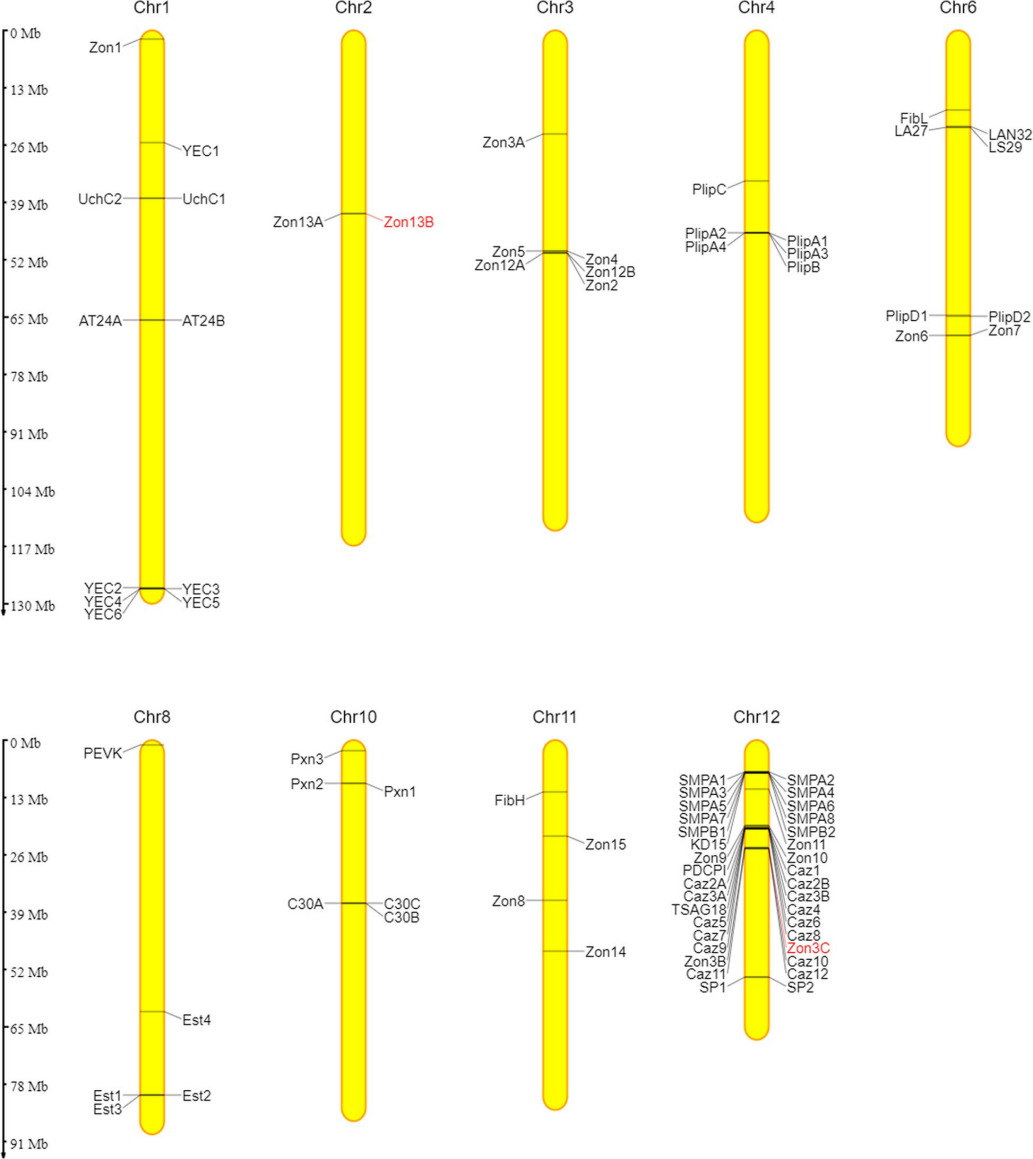
Localization of genes encoding silk proteins on the chromosomes of *L. lunatus*. It is noteworthy that the genes tend to form clusters with their paralogs; in particular, chromosome 12 contains large clusters of duplicated genes. The red color of the labeling indicates likely non-functional copies. The image was created with the online tool MG2C

By mapping our RNA-seq reads to the genomic sequence, we manually curated and annotated the cDNA sequences. We were also able to elucidate the exon –intron structures of individual genes (Fig. 5). The size of the silk genes ranges from rather large (over 30 kb in length), such as *fibH*, to rather small (less than 2 kb), such as a few *SMP* genes. However, most silk genes have an average size of 5 kb –10 kb. Since the candidate silk genes often appear to be present in multiple paralogs in clusters, we used the genome sequence of *L. lunatus* to localize them to chromosomes using the online tool MG2C (Fig. 6) [14]. The results revealed that although the candidate silk genes are located on nine different chromosomes, 41% of them reside on chromosome 12. In addition, we found that alternative splicing is relatively rare in the silk genes of *L. lunatus*; we identified only two alternative first exons in the *pxn1* gene, with both having a signal peptide for secretion.

### Properties of genes coding for important silk proteins

Overall, these putative proteins include homologs of standard silk components such as fibroins (FibH, FibL) and zonadhesin-like proteins (Zons) as well as novel protein and peptide families, including a large family of repetitive proteins with a putative adhesive function reminiscent of pseudofibroins from *P. conspersa*, which we have named cadhesins (Cazs) and two families of tyrosine-rich peptides (YEC and SMP). In addition, a few secretory enzymes and several other proteins with unknown function are found in the silk of *L. lunatus*. Thus, genes encoding putative silk proteins can be categorized into at least six different groups based on their similarity to known genes, their putative function, and/or their position in the genome (Fig. 3).

Group A, the fibroin group contains only two genes that include relatively conserved regions encoding the FibH and FibL chains and are located on chromosomes 11 and 6, respectively. Protein FibH is a large hydrophilic molecule comprising a repeating sequence of a 102 amino acid motif with scattered larger amino acids that lack the larger polyalanine or alanine-glycine stretches characteristic of crystalline regions (Figure S1). At both ends of the FibH are unique sequences that are relatively conserved (Figure S2). The gene encoding the putative FibL of *L. lunatus* has six exons and reveals approximately 35% and 50% identity to that of *B. mori* and *P. conspersa*, respectively.

Group B, the Caz group comprises 15 heterogeneous genes. They are located on chromosome 12 and are distributed in two different clusters, *caz1-9* and *caz10-12*, with a spatial distance of over 4 Mb between them. Another, slightly different member of this group, *TSAG18*, is located between *caz3B* and *caz4* (Fig. 4). Most *caz* genes have two-exon structures. The first exon is always short and encodes part of the signal peptide, while the second exon is rather long and often contains repetitive sequences. Therefore, the *caz* genes have a similar structure to *fibH*, but they are shorter and do not have a conserved sequence encoding Cys residues at their 3’ ends. Genes *caz1*, *caz8,* and *caz9* have an additional exon in the 3'-UTR. As mentioned above, Caz proteins contain repeated sequences with different types of repeats, mostly with a high proportion of Ser and Thr residues (Table 1). This is reminiscent of some Lepidoptera sericins and a few adhesion proteins in other organisms, although the similarity of some of their motifs is likely the result of convergent evolution rather than conservation. Alignments showing the similarity of Caz1 and Caz7 to known adhesion proteins with Ser- and Thr-rich motifs are depicted in Fig. S3.

Group C, the largest group, comprises 20 genes encoding the zonadhesin-like protein (Zons) family, which are highly heterologous and have their relatives in moths and caddisfly silk [1, 15]. They are rich in Cys residues and contain conserved EGF2 domains that are likely to be involved in protease inhibition. A few genes, such as *zon3C* with a stop codon in the second coding exon and *zon13B* with a frameshift-causing deletion in the second coding exon, are unlikely to produce proteins. Table S3 illustrates the main differences between them in terms of size, number of exons and number of EGF2 domains.

The chromosomal distribution of the *zon* genes in *L. lunatus* reveals that they occur in five different clusters. In particular, *zon4, zon5, zon12B, zon12A* and *zon2* are located on chromosome 3, while the trio of *zon9*, *zon10* and *PDCPI (PDCPI* is closely related to *zon9* and *zon10*) together with the pair *zon3B* and *zon3C* is found on chromosome 12. In addition, the pair *zon13A* and *zon13B* is located on chromosome 2, and *zon6* and *zon7* are located on chromosome 6. Six other individual zonadhesin-like genes are distributed on four different chromosomes: *zon1* on chromosome 1, *zon3A* on chromosome 3 (probably translocated from the *zon3B* + *C* cluster on chromosome 12), and *zon8, zon14* and *zon15* on chromosome 11, with *zon11* located on chromosome 12 (Fig. 5). The arrangement of the *zon* genes suggests that multiple duplications have occurred relatively recently, which is supported by the similarity of genes within the same cluster (e.g. *zon9* + *10* and *PDCPI* or *zon12A* + *B*) (Fig. S4a).

Group D, another large group, contains genes for 17 putative secretory enzymes—eight of which are pancreatic lipase-related proteins (Plip), four are putative esterases (Est), three are peroxinectins (Pxn) and at least two are potential serine proteases (SP). Interestingly, most of the genes encoding enzymes of *L. lunatus* are arranged in clusters, including five genes for lipases on chromosome 4 and a pair on chromosome 6 as well as a triplet of peroxinectins on chromosome 10 and a triplet of esterases on chromosome 8.

**Fig. 7:**
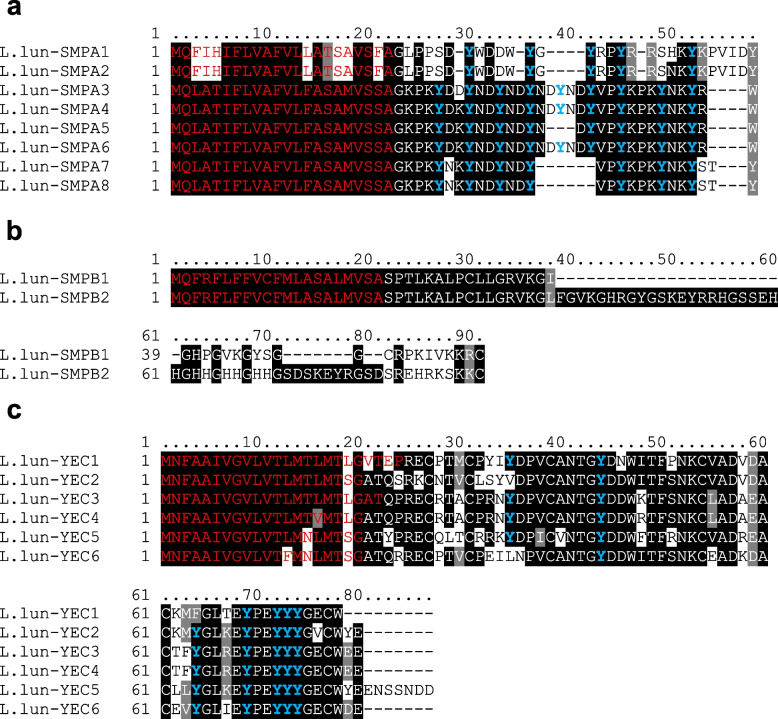
Alignments of small multicopy peptides found in *L. lunatus*. Signal peptides are indicated in red letters, conserved Tyr residues are highlighted in blue. **a**—small multicopy peptides A; **b**—small multicopy peptides B; **c**—small YEC-rich multicopy peptides

Group E consists of two families of genes for putative peptides with unknown function. We have named the first family YEC-rich multicopy peptides. There are six members of the YEC family on chromosome 1 (five of them are located in a tight cluster, see Fig. 5 and 6). The second family, called small multicopy peptides (SMPs), comprise 10 members whose genes are clustered on chromosome 12. SMPs are rather small and have a length of 49–91 amino acids, including the signal peptide. They can be divided into two subfamilies: the shorter SMPAs and the slightly longer SMPBs with eight and two members, respectively. SMPAs contain regularly spaced Tyr residues, whereas the Tyr residues in YEC are mostly grouped near the C-terminus (Fig. 7).

Group F, the last group is highly heterologous and consists of the genes for 12 remaining putative secretory proteins and peptides of unknown function. A few of them have homologs in other species, such as *PEVK*, which was first discovered in *H. occidentalis* [8] and whose ortholog in *L. lunatus* is located on chromosome 8. Other members are located on other chromosomes, and most of them have been named after prominent amino acids. These include genes for three C-rich 30 kDa proteins (C30s) on chromosome 10, two genes for paralogous proteins named AT24A and B (AT-rich 24 kDa proteins A and B) on chromosome 1, and a triplet of *LAN32*, *LA27* and *LS29* on chromosome 6. The gene encoding KD15 (KD-rich 15 kDa protein) is located on chromosome 12, near the *SMP* cluster. Finally, there are two paralogs of an uncharacterized CG3556-like protein (UchC1/2), which are encoded by two neighboring genes on chromosome 1 and have homologs in several insect orders.

## Discussion

We utilized a combination of transcriptome analysis, mapping of reads to a reference genome and identification of silk proteins by peptide mass fingerprinting to identify all silk proteins in *L. lunatus*, which belongs to the case-forming Trichoptera family (suborder Integripalpia). We identified over eighty putative silk proteins, characterized the structure of their genes, checked the tissue specificity of their expression and compared the results with those of the web spinning *P. conspersa*, a previously described predatory species from the caddisfly suborder Annulipalpia.

### MSG and PSG are not distinct in *L. lunatus*

The SGs of *L. lunatus* are located below the digestive tract on the ventral side of the body. In contrast to *L. lunatus*, the SG of *P. conspersa* are located above the digestive tract [1]. Both the silks of *L. lunatus* and *P. conspersa* contain a large proportion of housekeeping gene products. This suggests that the apocrine mechanism of silk secretion is similar to that of Lepidoptera [16].

Although the PSG, MSG and ASG are morphologically well separated in Lepidoptera, such clarity is absent in the SGs of Trichoptera. Previous studies on various trichopteran species, including *Stenopsyche marmorata* (suborder Annulipalpia, family Stenopsychidae) [17], *P. conspersa* (suborder Annulipalpia, family Polycentropodidae) [1], *Rhyacophila rougemonti* (suborder Integripalpia, family Rhyacophilidae) [11], *Hydroptila aegyptia* (suborder Integripalpia, family Hydroptilidae) [11], and *Leptocerus tineiformis* (suborder Integripalpia, family Leptoceridae) [11] have shown that there is no clear boundary between the MSG and the PSG. In *L. lunatus*, we were also unable to establish a clear boundary and therefore collectively referred to them as rear SGs.

Although the lack of morphological distinction between MSG and PSG in seems universal, it is less clear whether they are also functionally undifferentiated. For example, histological sections in *P. conspersa* revealed a potential functional division within the posterior segment of the SG. An uncoated fibroin core forms at its rearmost end, while the coating gradually builds up towards ASG [1]. Similarly, Hatano and Nagashima (2015) distinguished the MSG and PSG in the rear SG of *S. marmorata*, considering the posteriormost, very short part of the SG as the PSG. This part produces a thin axial thread of unknown composition located in the middle of fibroin filament [17]. This may be a peculiarity of a small group of species because such axial thread has not been observed in any other species [1, 10]. Remarkably, the proposed MSG in *S. marmorata* seems to produce both fibroin and the adhesive coating [17]. Another study by Kim et al. (2020) investigated the morphology of SGs using electron microscopy in the limnephilid caddisfly *Hydatophylax nigrovittatus* [10]. They divided the SG into ASG and PSG and found that both parts contain different secretory cells. The epithelium of the ASG contains fine granular materials as secretory products in contrast to the large secretory globules found in the rear SG. Thus, Kim et al. (2020) hypothesized that the rear SG produces fibroin, while the ASG secretes sticky substances required for gluing together the two silk filaments [10].

However, our staining of the transverse section through the *L. lunatus* larval body reveals that the stored proteins in the SG lumen form two layers that can be distinguished throughout the entire rear SG, extending from the rearmost part. As depicted in Fig. 1, there is a thin, blue-colored peripheral coating covering the thick, purple-colored core layer. This indicates that at least part of this outer layer is formed in the rear SG. The remainder of the outer layer is then formed in the ASG. Our expression analysis revealed that there are several transcripts specific to the ASG. The ASG produces the enzymes lipases and proteases as well as two copies of the alanine- and threonine-rich protein AT24 (Table 2). The AT24s are putative 24 kDa nonrepetitive proteins with unknown function. It appears that similar sequences are also present in other Integripalpia caddisflies. It will be interesting to investigate the role of AT24 in adhesion.

The PSGs of Lepidoptera produce a silk core of fibroins (FibH and FibL). Previous data on *P. conspersa* revealed that transcripts for most of the studied proteins, including FibH and FibL are expressed in both MSG and PSG [1]. Consistently, in *L. lunatus*, both fibroin subunits are also produced throughout the entire rear SG, along with numerous other silk components. At least a few of these proteins are located in the axial fiber—for example, the PEVK-like protein in Hesperophylax *occidentalis* was identified in the axial filament and not in the adhesive outer layer [8]. However, a number of these proteins are probably a part of the adhesive coating. In particular, Caz proteins are likely candidates as they contain motifs resembling those in lepidopteran sericins (Fig. S3). Histological evidence suggests that fibroins and adhesives in the same SG compartment form separate layers without mixing. This is consistent with previous results by Hatano and Nagashima (2015) [17], who showed that the secretory fibroin globules in *S. marmorata* penetrate through the outer silk layer and accumulate in the middle silk gland lumen.

It is therefore obviousthat the model of silk fiber assembly initiated by the synthesis of a hydrophobic insoluble fibroin core in PSG to which soluble coatings in MSG are progressively added is not applicable to all Trichoptera.

### Individual silk genes and their products

*FibH* is the largest gene identified in the silk of *L. lunatus*. Similar to other known fibroin genes of caddisflies and moths, it is structured in two exons. The substantial size of the FibH protein (almost 1000 kDa) appears to be characteristic of caddisfly fibroins [18] and may be important for mechanical function, possibly compensating for its lower crystallinity compared to Lepidoptera. The sequences of the *L. lunatus* fibroin differ from moth fibroins in terms of their hydrophilicity and lack of poly-Ala or Ala-Gly repeats that can form beta-sheets. Instead, it has been suggested that the FibH molecules in caddisfly silk fibers are interconnected through serine phosphorylation in combination with Ca^2+ ^[4]. A high level of serine phosphorylation of (SX)n motifs in caddisfly fibroins has been described in caddisfly species as distant as *Parapsyche elsis* (Annulipalpia) and *Hesperophylax* sp. (Integripalpia) [12]. The latter belongs to the tribe Limnephilini, which makes it closely related to *L. lunatus*.

Another important mechanism that ensures the strength of silk in water is dityrosine cross-linking by the enzymes peroxinectins (peroxidases). Peroxinectin has been shown to catalyze dityrosine formation in the sticky underwater silk of the caddisfly larva *H. occidentalis* [8]. We found a number of proteins and peptides, including SMPs, containing high levels of tyrosine residues that may serve as substrates for the cross-linking process.

We found putative homologs of several proteins previously discovered in other limnephilid caddisflies (Table 2), including Pxn and PEVK—found in *H. occidentalis* [8]*.* The silk of *L. lunatus* also contains at least five putative lipases, whose role is unclear. Lipases have also been found in the silk of certain moths, including *Tineola bisselliella* and *Galleria mellonella,* and may represent enzymes that fulfill an original digestive function in salivary glands [16, 19, 20]. In addition, the lipase in *B. mori* plays a role in defense mechanisms as a physiological barrier against *B. mori* nuclear polyhedrosis virus (BmNPV) at the site of viral infections [21]. Furthermore, the role of conserved silk gland-specific protease is also unknown. Its homolog in *T. bisselliella* was confirmed as an SG-specific transcript by qPCR [18]. Another protein of unknown function, LAN32 (originally found in *P. conspersa*) [1], was found to have homologs conserved in all the examined Trichoptera (Table 2).

### Multiple duplications of silk genes in *L. lunatus* compared to those in *P. conspersa*

The silk proteins of both *L. lunatus* and *P. conspersa* can be divided into the same six categories (Fig. 3). However, the number of different proteins found in *L. lunatus* is almost three times higher than the number of proteins previously found in *P. conspersa*, a caddisfly of the suborder Annulipalpia [1], which was analyzed utilizing the same approach. The greatest difference in the number of detected gene products between *L. lunatus* and *P. conspersa* lies in the class of small multicopy peptides (YECs and SMPs), which has almost 20 members in *L. lunatus* or *L. flavicornis*. In contrast, there is only one peptide in *P. conspersa* that is reminiscent of this family by its size and the presence of aromatic amino acids. However, it is unclear whether this is a true homolog of the SMPs or YECs of *L. lunatus*, as no match was found at the amino acid sequence level.

Further, the proteins found in the silk of *L. lunatus* include 14 cadhesins. One of these is called Caz12 belonging to the smaller *Caz* gene cluster on chromosome 12, and appears to be a homolog of the SGA28 protein in *P. conspersa* [1] and the S.ang7.801.2 protein from *Stenopsyche angustata* [2] (Table 2). The protein SGA28 was previously localized in the genome of *P. conspersa* within a small group of genes called pseudofibroins [1]. Thus far, *P. conspersa* has five described pseudofibroin genes, which resemble the cadhesins of *L. lunatus* in terms of the arrangement of the exons and repetitiveness. However, in contrast to *L. lunatus* Cazs, the pseudofibroins of *P. conspersa* contain a significant proportion of Ala residues, similarly to *P. conspersa*FibH [1].

It appears that the overrepresented genes in *L. lunatus* are localized in clusters, thereby suggesting a possible result of recent duplications. In addition, *zons* and *plips* that occur at multiple loci in the genome, tend to be more similar to their paralogs within the same cluster as compared to those outside (Fig. S4)*.* This level of duplication could represent an adaptation of silk proteins to achieve higher adhesion strength compared to the free-floating filaments of retreats and trapping nets of caddisflies of the suborder Annulipalpia. Our results support the idea of increasing the structural complexity of silk in rigid case producers compared to cocoon and trap net builders.

## Conclusions

Our results challenge the conventional model of silk fiber formation based on results in Lepidoptera and reveal that the synthesis of a hydrophobic, insoluble fibroin core in the PSG is not applicable to Trichoptera. In particular, the SGs of *L. lunatus* lack a clear morphological boundary between the MSG and the PSG, collectively referred to as the rear SG. Furthermore, our study reveals that fibroin and adhesive proteins are simultaneously produced in the entire rear SG.

A comparative analysis with *P. conspersa* reveals an increase in the number of silk genes in *L. lunatus*, particularly in the class of small multicopy peptides (YECs and SMPs). Further, the overrepresented genes in *L. lunatus* are organized in clusters, thereby suggesting possible recent duplications that may represent an adaptation that contributes to higher adhesion strength and distinguishes it from caddisflies of the suborder Annulipalpia.

## Materials and methods

### Biological material

The last instar larvae of *L. lunatus L. flavicornis* and *P. conspersa* were collected in a stream approximately 7 km east of České Budějovice, in the Czechia (48°59′23.3″N, 14°33′55.3″E). Their species was verified by DNA barcoding—that is, sequencing of the cytochrome c oxidase I (COI) fragment barcode (Sequences accessible in GenBank with IDs PP092039, PP091968, and PP092106).

### Histology

The cuticle of larvae anesthetized with CO_2_ was punctured with a fine needle under a Bouin-Hollande fixative solution supplemented with mercuric chloride to allow penetration of the fixative. The samples were fixed overnight at 4 °C. Standard histological procedures were used for the dehydration of the tissue, embedding in Paraplast, sectioning (10 μm), deparaffinization, and rehydration. Sections were treated with Lugol's iodine solution followed by a 7.5% sodium thiosulfate solution to remove residual heavy metal ions, washed in distilled water, and stained with HT15 Trichrome staining Kit [22] (Masson) (Sigma-Aldrich, Inc., St. Louis, MO, USA) according to the manufacturer's protocol. The stained sections were dehydrated and mounted in DPX embedding medium (Fluka, Buchs, Switzerland). High-resolution images of the cross sections were acquired using the BX63 microscope, DP74 CMOS camera, and cellSens software (Olympus Corporation, Tokyo, Japan) by stitching multiple images and Z stack imaging.

### Ultrastructure of silk

The silk samples of *L. lunatus* were cut from their cases and glued to the surface of aluminum holders; the fibers of *P. conspersa* were obtained by letting the larvae spin a retreat in containers with aluminum holders on which the fibers sank upon water removal. The samples were subsequently coated with gold and analyzed using a Jeol JSM-7401F scanning electron microscope (Jeol, Akishima, Japan).

### RNA isolation and construction of transcriptomes

For the preparation of cDNA libraries, we prepared silk glands from anesthetized larvae of the last instar of *L. lunatus* and *L. flavicornis.* In addition, we isolated RNA using TRIzol reagent (Life Technologies, Carlsbad, USA) in accordance with the manufacturer’s protocol.

Further, we constructed cDNA libraries as described earlier [23], and used the MiSeq (Illumina, San Diego, CA, United States) instrument to produce 2 × 150 paired-end reads. The yield of the Illumina sequencing was never less than 16.8 × 10 [6] reads per sample. The transcriptomes were assembled using the Trinity software integrated in the Galaxy platform [24], as described earlier [25] The completeness of the transcriptomes was estimated using BUSCO (Galaxy Version 5.3.2 + galaxy0, database for Insecta) [26].

### Mapping the reads to a reference genome

We manually curated the transcript sequences of the candidate silk genes in *L. lunatus* against the available genomic sequences (GenBank:GCA_917563855.2) [27] using BLAST searches [28]. For easier distinguishing of exon–intron boundaries and visualization of genome clusters, we mapped the Illumina reads to the genome using RNA STAR software (Galaxy version 2.7.8a + galaxy0) in combination with IGV 2.9.4 [20]. We also constructed chromosomal maps using the MG2C online tool [14].

### Protein identification using mass spectrometry

We cleaned the spun silk sample of plant debris under a binocular microscope, dissolved in urea, trypsinized, and analyzed using nanoscale liquid chromatography coupled with tandem mass spectrometry (nLC-MS/MS), as previously described [19]. Peptide mass fingerprinting was performed using MaxQuant 1.6.17.0 software [29]. We used the default settings for false discovery rate (FDR) and minimum peptide length (i.e., 1% and seven amino acids, respectively). We searched raw files against our custom peptide database predicted from RNA sequencing.

### Quantification of transcript levels with quantitative RT-PCR

We used the following tissues for transcriptional analysis: intestine, head, thorax, and SGs. Further, we divided the SGs into three parts at the fold sites. The qRT-PCR was performed using HOT FIREPol EvaGreen qPCR Mix Plus (Solis BioDyne, Tartu, Estonia). The reaction volume of 20 µl contained 5 µl diluted cDNA and 250 nM primer. Amplification was performed on a Rotor-Gene Q MDx 2plex HRM (Qiagen, Hilden, Germany) for 40 cycles (95°C for 15 s; annealing temperature adapted to the primer pair for 30 s; 72°C for 20 s) after an initial denaturation/pole activation step (95°C for 15 min).

Primers (Table S4) were designed using the Lasergene PrimerSelect software (DNASTAR, Madison, USA). The resulting data were analyzed and quantified using Rotor Gene Q 2.3.5 software. Values were normalized to glyceraldehyde 3-phosphate dehydrogenase (GAPDH) transcript. Each sample was analyzed in triplicate. Further, statistical significance was determined using the Kruskal–Wallis rank sum test followed by the pairwise Wilcoxon test. The calculations were performed using R 4.2.2 in combination with RStudio 2022.12.0 [30, 31].

### Representation of the relationships among the protein sequences

Protein trees were created with the IQ-TREE online tool [32]. The models for tree construction were selected using the automatic search by ModelFinder [33] for the best substitution model with FreeRate heterogenity enabled. The branch support was calculated by the bootstrap analysis with 1000 repeats. The SH-aLRT branch test was disabled.

### Supplementary Information

Supplementary material 1.

## Data Availability

The experimental data supporting the results of this study are available in this article or in the supplementary materials. The raw data have been deposited in NCBI under the bioproject accession numbers PRJNA1075646 (*Limnephilus lunatus*) and PRJNA1075661 (*Limnephilus flavicornis*). List of silk gene candidates and their GenBank accession codes are shown in Table 1 and Supplementary Tables 1 and 2.
