# Supplementary material for: Comprehensive analysis of silk proteins and gland compartments in Limnephilus lunatus, a case-making trichopteran
Source: BMC Genomics. 2024 May 14;25:472. doi: 10.1186/s12864-024-10381-4 (PMC11092239; doi:10.1186/s12864-024-10381-4)
Supplement: Supplementary file 1 — Supplementary material 1. [file 12864_2024_10381_MOESM1_ESM.pdf]

Figure S1. FibH protein sequence predicted from the genomic region. The repeated segments in the central repetitive regions are aligned to visualize the regularity of the repeats. Amino acids are highlighted with cyan (hydrophilic: RK), magenta (hydrophobic: VLI).

```
MRAILLLIVFCSWQVHRTGSSAVSNKVQDLFKHGRHLDSDGLHERILEEEDVIETNSKGEIIEKIISRKEIISDDASDSWSDSDSSESGSTEQIIKQIIIVQ
ERPKHGHHHAKEKIYEEEIVIKKIGDVIHGVDVPLSIDGGLRRGGHSGRRGPKLSISKSISIEQVVRPSVYTKISRSSSVSVEGGRQRGPWGKGKGAWGSLG
GLSGSGDLDSLGGLGGLGGLRGPWGRRGKVSISRSASIERIVTPGVYTKISRSS
```

```
--------------------------------------------------SVSVEGGRRRGPWGRRGKVSISRSVSIERIVTPGVYTKISRSSSISVEGGRR
RGPWGYGRGLGGLIGSGDLDGLGGVGGLGGLGGRRGPWGRGYGSSGSVSVSVSVEGGRRRGPWGRRGKVSISRSVSIERIVTPGVYTKISRSSSVSVEGGRR
RGPWG----------------------------------RGYGSSGSVSVSVSVEGGRRRGPWGRRGKVSISRSVSIERIVTPGVYTKISRSSSVSVEGGRR
RGPWGYGRGLGGLSGSGDLDGLGGVGGLGGLGGRRGPWGRGYGSSGSVSVSVSVEGGRRRGPWGRRGKVSISRSVSIERIVTPVVYTKISRSSSVSVEGGRR
RGPWGYGRGLGGLSGSGDLDGLGGLGGLGGLGGRRGPWGRGYGSSGSVSVSVSVEGGRRRGPWGRRGKVSISRSVSIERIVTPGVYTKISRSSSVSVEGGRR
RGPWGYGRGLGGLSGSGDLDGLGGVDGLGGLGGRRGPWGRGYGSSGSVSVSVSVEGGRRRGPWGRRGKVSISRSVSIERIVTPGVYTKISRSSSVSVEGGRR
RGPWGYGRGLGGLSGSGDLDGLGGVSGLGGLGGRRGPWGRGYGSSGSVSVSVSVEGGRRRGPWGRRGKVSISRSVSIERIVTPGVYTKISRSSSVSVEGGRR
RGPWGYGRGLGGLSGSGDLDGLGGVGGLGGLGGRRGPWGRGYGSSGSVSVSVSVEGGRRRGPWGRRGKVSISRSVSIERIVTPGVYTKISRSSSVSVEGGRR
RGPWGYGRGLGGLSGSGDLDGLGGVGGLGGLGGRRGPWGRGYGSSGSVSVSVSVEGGRRRGPWGRRGKVSISRSVSIERIVTPGVYTKISRSSSVSVEGGRR
RGPWGYGRGLGGLSGSGDLDGLGGVGGLGGLGGRRGPWGRGYGSSGSASVSVSVEGGRRRGPWGRRGKVSISRSVSIERIVTPGVYTKISRSSSVSVEGGRR
RGPWGYGRGLGGLSGSGDLDGLGGVGGLGGLGGRRGPWGRGYGSSGSVSVSVSVEGGRRRGPWGRRGKVSISRSVSIERIVTPGVYTKISRSSSVSVEGGRR
RGPWGYGRGLGGLSGSGDLDGLGGVGGLGGLGGRRGPWGRGYGSSGSASVSVSVEGGRRRGPWGRRGKVSISRSVSIERIVTPGVYTKISRSSSVSVEGGRR
RGPWGYGRGLGRLSGSGDLDGLGGVGGLGGLGGRRGPWGRGYGSSGSVSVSVSVEGGRRRGPWGRRGKVSISRSVSIERIVTPGVYTKISRSSSVSVEGGRR
RGPWGYGRGLGGLSGSGDLDGLGGVGGLGGLGGRRGPWGRGYGSSGSASVSVSVEGGRRRGPWGRRGKVSISRSVSIERIVTPGVYTKISRSSSVSVEGGRR
RGPWGYGRGLGGLSGSGDLDGLGGVGGLGGLGGRRGPWGRGYGSSGSVSVSVSVEGGRRRGPWGRRGKVSISRSVSIERIVTPGVYTKISRSSSVSVEGGRR
RGPWGYGRGLGGLSGSGDLDGLGGVGGLGGLGGRRGPWGRGYGSSGSVSVSVSVEGGRRRGPWGRRGKVSISRSVSIERIVTPGVYTKISRSSSVSVEGGRR
RGPWGYGRGLGGLSGSGDLDGLGGVGGLGGLGGRRGPWGRGYGSSGSVSVSVSVEGGRRRGPWGRRGKVSISRSVSIERIVTPGVYTKISRSSSVSVEGGRR
RGPWGYGRGLGGLSGSGDLDGLGGVGGLGGLGGRRGPWGRGYGSSGSVSVSVSVEGGRRRGPWGRRGKVSISRSVSIERIVTPGVYTKISRSSSVSVEGGRR
RGPWGYGRGLGGLSGSGDLDGLGGVGGLGGLGGRRGPWGRGYGSSGSVSVSVSVEGGRRRGPWGRRGKVSISRSVSIERIVTPGVYTKISRSSSVSVEGGRR
RGPWGYGRGLGGLSGSGDLDGLGGVGGLGGLGGRRGPWGRGYGSSGSASVSVSVEGGRRRGPWGRRGKVSISRSVSIERIVTPGVYTKISRSSSVSVEGGRR
PGPWGYGRGLGGLSGSGDLDGLGGVGGLGGLGGRRGPWGRGYGSSGSVSVSVSVEGGRRRGPWGRRGKVSISRSVSIERIVTPGVYTKISRSSSVSVEGGRR
RGPWGYGRGLGGLSGSGDLDGLGGVGGLGGLGGRRGPWGRGYGSSGSVSVSVSVEGGRRRGPWGRRGKVSISRSVSIERIVTPGVYTKISRSSSVSVEGGRR
RGPWGYGRGLGGLSGSGDLDGLGGVGGLGGLGGRRGPWGRGYGSSGSASVSVSVERGRRRGPWGRRGKVSISRSVSIERIVTPGVYTKISRSSSVSVEGGRR
RGPWGYGRGLGGLSGSGDLDGLGGVSGLGGLGGRRGPWGRGYGSSGSVSVSVSVEGGRRRGPWGRRGKVSISRSVSIERIVTPGVYTKISRSSSVSVEGGRR
RGPWGYGRGLGGLSGSGDLDGLGGVGGLGGLGGRRGPWGRGYGSSGSESVSVSVEGGRRRGPWGRRGKVSISRSVSIERIVTPGVYTKISRSSSVSVEGGRR
RGPWG----------------------------------RGYGSSGSVSVSVSVEGGRRRGPWGRRGKVSISRSVSIERIVTPGVYTKISRSSSVSVEGGRR
RGPWGYGRGLGGLSGSGDLDGLGGVGGLGGLGGRRGPWGRGYGSSGSVSVSVSVEGGRRRGPWGRRGKVSISRSVSIERIVTPGVYTKISRSSSVSVEGGRR
RGPWGYGRGLGGLSGSGDLDGLGGVGGLGGLGGRRGPWGRGYGSSGSVSVSVSVEGGRRRGPWGRRGKVSISRSVSIERIVTPGVYTKISRSSSVSVEGGRR
RGPWGYGRGLGGLSGSGDLDGLGGVGGLGGLGGRRGPWGRGYGSSGSVSVSVSVEGGRRRGPWGRRGKVSISRSVSIERIVTPGVYTKISRSSSVSVEGGRR
RGPWG----------------------------------RGYGSSGSVSVSASVEGGRRRGPWGRRGKVSISRSVSIERIVTPGVYTKISRSSSVSVEGGRR
RGPWGYGRGLGGLSGSGDLDGLGGVGGLGGLGGRRGPWGRGYGSSGSVSVSVSVEGGPRRGPWGRRGKVSISRSVSIERIVTPGVYTKISRSSSVSVEGGRR
RGPWGYGRGLGGLSGSGDLDGLGGVGALGGLGGRRGPWGRGYGSSGSVSVSVSVEGGRRRGPWGRRGKVSISRSVSIERIVTPGVYTKISRSSSVSVEGGRR
RGPWGYGRGLGGLSGSGDLDGLGGVGGLGGLGGRRGPWGRGYGSSGSVSVSVSVEGGRRRGPWGRRGKVSISRSVSIERIVTPGVYTKISRSSSVTVEGGRR
RGPWGYGRGLGGLSGSGDLDGLGGVGGLGGLGGRRGPWGRGYGSSGSVSVSVSVEGGRRRGPWGRRGKVSISRSVSIERIVTPGVYTKISRSSSVSVEGGRR
RGPWGYGQGLGGLSGSGDLDGLGGVGGLGGLGGRRGPWGRGYGSSGSASVSVSVEGGRRRGPWGRRGKVSISRSVSIERIVTPGVYTKISRSSSVSVEGGRR
RGPWGYGRGLGGLSGSGDLDGLGGVGGLGGLGGRRGPWGRGYGSSGSVSVSVSVEGGRRRGPWGRRGKVSISRSVSIERIVTPGVYTKISRSSSVSVEGGRR
RGPWGYGRGLGGLSGSGDLDGLGGVGSLGGLGGRRGPWGRGYGSSGSVSVSVSVEEGRRRGPWGRRGKVSISRSVSIERIVTPGVYTKISRSSSVSVEGGRR
RGPWGYGRGLGGLSGSGDLDGLGGVGGLGGLGGRRGPWGRGYGSSGSASVSVSVEGGRRRGPWGRRGKVSISRSVSIERIVTPGVYTKISRSSSVSVEGGRR
RGPWGYGRGLGGLSGSGDLDGLGGLGGLGGLGGRRGPWGRGYGSSGSASVSVSVEGGRRRGPWGRRGKVSISRSVSIERIVTPGVYTKISRSSSVSVEGGRR
RGPWGYGRGLGGLSGSGDLDGLGGVSGLGGLGGRRGPWGRGYGSSGSVSVSVSVEGGRRRGPWGRRGKVSISRSVSIERIVTPGVYTKISRSSSVSVEGGRR
RGPWGYGRGLGGLSGSGDLDGLGGVGGLGGLGGRRGPWGRGYGSSGSESVSVSVEGGRRRGPWGRRGKVSISRSVSIERIVTPGVYTKISRSSSVSVEGGRR
RGPWG----------------------------------RGYGSSGSVSVSVSVEGGRRRGPWGRRGKVSISRSVSIERIVTPGVYTKISRSSSVSVEGGRR
RGPWGYGRGLGGLSGSGDLDGLGGVGGLGGLGGRRGPWGRGYGSSGSVSVSVSVEGGRRRGPWGRRGKVSISRSVSIERIVTPGVYTKISRSSSVSVEGGRR
RGPWGYGRGLGGLSGSGDLDGLGGVGGLGGLGGRRGPWGRGYGSSGSVSVSVSVEGGRRRGPWGRRGKVSISRSVSIERIVTPGVYTKISRSSSVSVEGGRR
RGPWGYGRGLGGLSGSGDLDGLGGVGGLGGLGGRRGPWGRGYGSSGSVSVSVSVEGGRRRGPWGRRGKVSISRSVSIERIVTPGVYTKISRSSSVSVEGGRR
RGPWG----------------------------------RGYGSSGSVSVSASVEGGRRRGPWGRRGKVSISRSVSIERIVTPGVYTKISRSSSVSVEGGRR
RGPWGYGRGLGGLSGSGDLDGLGGVGGLGGLGGRRGPWGRGYGSSGSVSVSVSVEGGPRRGPWGRRGKVSISRSVSIERIVTPGVYTKISRSSSVSVEGGRR
RGPWGYGRGLGGLSGSGDLDGLGGVGALGGLGGRRGPWGRGYGSSGSVSVSVSVEGGRRRGPWGRRGKVSISRSVSIERIVTPGVYTKISRSSSVSVEGGRR
RGPWGYGRGLGGLSGSGDLDGLGGVGGLGGLGGRRGPWGRGYGSSGSVSVSVSVEGGRRRGPWGRRGKVSISRSVSIERIVTPGVYTKISRSSSVTVEGGRR
RGPWGYGRGLGGLSGSGDLDGLGGVGGLGGLGGRRGPWGRGYGSSGSVSVSVSVEGGRRRGPWGRRGKVSISRSVSIERIVTPGVYTKISRSSSVSVEGGRR
RGPWGYGQGLGGLSGSGDLDGLGGVGGLGGLGGRRGPWGRGYGSSGSASVSVSVEGGRRRGPWGRRGKVSISRSVSIERIVTPGVYTKISRSSSVSVEGGRR
RGPWGYGRGLGGLSGSGDLDGLGGVGGLGGLGGRRGPWGRGYGSSGSVSVSVSVEGGRRRGPWGRRGKVSISRSVSIERIVTPGVYTKISRSSSVSVEGGRR
RGPWGYGRGLGGLSGSGDLDGLGGVGSLGGLGGRRGPWGRGYGSSGSVSVSVSVEEGRRRGPWGRRGKVSISRSVSIERIVTPGVYTKISRSSSVSVEGGRR
RGPWGYGRGLGGLSGSGDLDGLGGVGGLGGLGGRRGPWGRGYGSSGSASVSVSVEGGRRRGPWGRRGKVSISRSVSIERIVTPGVYTKISRSSSVSVEGGRR
```

```
RGPWGYGRGLGGLSGSGDLDGLGGLGGLGGLGGRRGPWGRGYGSSGSASVSVSVEGGRRRGPWGRRGKVSISRSVSIERIVTPGVYTKISRSSSVSVEGGRR
RGPWGYGQGLGGLSGSGDLDGLGGVGGLGGLGGRRGPWGRGYGSSGSASVSVSVEGGRRRGPWGRRGKVSISRSVSIERIVTPGVYTKISRSSSVSVEGGRR
RGPWGYGRGLGGLSGSGDLDGLGGVGGLGGLGGRRGPWGRGYGSSGSVSVSVSVEGGRRRGPWGRRGKVSISRSVSIERIVTPGVYTKISRSSSVSVEGGRR
RGPWGYGRGLGGLSGSGDLDGLGGVGGLGGLGGRRGPWGRGYGSSGSVSVSVSVEEGRRRGPWGRRGKVSISRSVSIERIVTPGVYTKISRSSSVSVEGGRR
RGPWGYGRGLGGLSGSGDLDGLGGVGGLGGLGGRRGPWGRGYGSSGSASVSVSVEGGRRRGPWGRRGKVSISRSVSIERIVTPGVYTKISRSSSVSVEGGRR
RGPWGYGRGLGGLSGSGDLDGLGGVGGLGGLGGRRGPWGRGYGSSGSVSVSV--EGGRRRGPWGRRGKVSISRSVSIERIVTPGVYTKISRSSSVSVEGGRR
RGPWGYGRGLGGLSGSGDLDGLGGVGGLGGLGGRRGPWGRGYGSSGSVSVSVSVEGGRRRGPWGRRGKVSISRSVSIERIVTPGVYTKISRSSSVSVEGGRR
RGPWGYGRGLGGLSGSGDLDGLGGVGGLGGLGGRRGPWGRGYGSSGSASVSVSVEGGRRRGPWGRRGKVSISRSVSIERIVTPGVYTKISRSSSVSVEGGRR
RGPWGYGRGLGGLSGSGDLDGLGGVGGLGGLGGRRGPWGRGYGSSGSVSVSVSVEGGRRRGPWGRRGKVSISRSVSIERIVTPGVYTKISRSSSVSVEGGRR
RGPWGYGRGLGGLSGSGDLDGLGGVGGLGGLGGRRGPWGRGYGSSGSVSVSVSVEGGRRRGPWGRRGKVSISRSVSIERIVTPGVYTKISRSSSVSVEGGRR
RGPWGYGRGLGGLSGSGDLDGLGGVGGLGGLGGRRGPWGRGYGSSGSVSVSVSVEGGRRRGPWGRRGKVSISRSVSIERIVTPGVYTKISRSSSVSVEGGRR
RGPWGYGRGLGGLSGSGDLDGLGGVGGLGGLGGRRGPWGRGYGSSGSVSVSVSVEGGRRRGPWGRRGKVSISRSVSIERIVTPGVYTKISRSSSVSVEGGRR
RGPWGYGRGLGGLSGSGDLDGLGGVGGLGGLGGRRGPWGRGYGSSGSASVSVSVEGGRRRGPWGRRGKVSISRSVSIERIVTPGVYTKISGSSSVSVEGGRR
RGPWG----------------------------------RGYGSSGSVSVSVSVEGGRRRGPWGRRGKVSISRSVSIERIVTPGVYTKISRSSSVSVEGGRR
RGPWGYGRGLGGLSGSGDLDGLGSVGGLGGLGGRRGPWGRGYGSSGSVSVSVSVEGGRRRGPWGRRGKVSISRSVSIERIVTPGVYTKISRSSSVSVEGGRR
RGPWGYGRGLGGLSGSGDLDGLGGVGGLGGLGGRRGPWGRGYGSSGSVSVSVSVEGGRRRGPWGRRGKVSISRSVSIERIVTPGVYTKISRSSSVSVEGGRR
RGPWGYGRGLGGLSGSGDLDGLGGLGGLGGLGGRRGPWGRGYGSSGSASVSVSVEGGRRRGPWGRRGKVSISRSVSIERIVTPGVYTKISRSSSVSVEGGRR
RGPWGYGRGLGGLSGSGDLDGLGGLGGLGGLGGRRGPWGRGYGSSGSVSVSVSVEGGRRRGPWGRRGKVSISRSVSIERIVTPGVYTKISRSSSVSVEGGRR
RGPWGYGRGLGGLSGSGDLDGLGGLGGLGGLGGRRGPWGRGYGSSGSASVSVSVEGGRRRGPWGR-------------------------------------
----------------------------------------GYGSSGSVSVSVSIERGIRRGPWGRRGKVSISRSVSIERIVIPGVYTKISRSSSVSVEGERR
RGPWGYGRGLGGLSGSGDLDGLGGLGGLGGLGGRRGPWGRGYGSSGSVSVSVSVEGGRRRGPWGRRGKVSISRSVSIERIVTPGVYTKISRSSSVSVEGGRR
RVPWGYGRGLGGLSGSGDLDGLGGLGGLGGLGGRRGPWGRGYGSSGSASVSVSVEGGRRRGPWGR-------------------------------------
----------------------------------------GYGSSGSVSVSVSIERGIRRGPWGRRGKVSISRSVSIERIVTPGVYTKISRSSSVSVEGGRR
RGPWGYGRGLGGLSGSGDLDGLGGLGGLGGLGGRRGPWGRGYGSSGSVSVSVSVEGGRRRGPWGRRGKVSISRSVSIERIVTPGVYTKISRSSSVSVEGGRR
QGPWG----------------------------------RGYGSSGSVSVSVSVEGGRRRGPWGRRGKVSISRSVSIERIVTPGVYTKISRSSSVSVEGGRR
RGPWGYGRGLGGLSGSGDLDGLGGVGGLGGLGGRRGPWGRGYGSSGSVSVSVSVEGGRRRGPWGRRGKVSISRSVSIERIVTPGVYTKISRSSSVSVEGGRR
RGPWGYGRGLGGLSGSGDLDGLGGLGGLGGLGGRRGPWGRRYGSSGSASVSVSVEGGRRRGPWGR-------------------------------------
----------------------------------------GYGSSGSVSVSVSIERGIRRGPWGRRGKVSISRSVSIERIVTPGVYTKISRSSSVSVEGGRR
RGPWGYGRGLGGVSGSGDLDGLGGLGGLGGLGGRRGPWGRGYGSSGSASVSVSVEGGRRRGPWGR-------------------------------------
----------------------------------------GYGSSGSVSVSVSIERGIRRGPWGRRGKVSISRSVSIERIVTPGVYTKISRSSSVSVEGGRR
RGPWGYGRGLGGLSGSGDLDGLGGLGGLGGLGGRRGPWGRGYGSSGSASVSVSIERGIRRGPWGRRGKVSISRSVSIERIVTPGVFTKISRSSSVSVEGGRR
RGPWGYGRGLGGLSGSGDLDGLGGLGGLGGLGGRRGPWGRGYGSSGSASVSVSIERGIRRGPWGRRGKVSISRSVSIERIVTPGVYTKISRSSSVSVEGGRR
RGPWGYGRGLGGLSGSGDLDGLGGLGGLGGLGGRRGPWGRGYGSSGSVSVSVSIERGIRRGPWGRRGKVSISRSVSIERIVTPGVYTKISRSSSVSVEGGRR
RGPWG-----------------------------------------------------------RRGKVSISRSVSIERIVTPGVYTKISRSSSVSVEGGRR
RGPWGYGRGLGGVSGSGDLDGLGGLGGLGGLGGR--------------------------------------------------------------------
RGPWGYGRGVGGVSGSGDLDGLGGLGGLGGLGGRRGPWGRGYGSSGSASVSVSVEGGRR-------------------------------------------
RGPWG----------------------------------RGYGSSGSVSVSVSIERGIRRGPWGRRGKVSISRSVSIERIVTPGVYTKISRSSSVSVEGGRR
RGP--------------------GLGGLGGLGGRRGPWGRGYGSSGSVSVSVSIERGIRRGPWGRRGKVSISRSVSIERIVTPGVYTKISRSSSVSVEGGRR
RGPWGYGRGLGGLSGSGDLDGLGGLGGLGGLGGRRGPWGRGYGSSGSVSVSVSVEGGRRRGPWGRRGKVSISRSVSIERIVTPGVYTKISRSSSVSVEGGRR
RGPWG----------------------------------RGYGSSGSVSVSVSVEGGRRRGPWGRRGKVSISRSVSIERIVTPGVYTKISRSSSVSVEGGRR
RGPWGYGRGLGGLSGSGDLDGLGGVGGLGGLGGRRGPWGRGYGSSGSVSVSVSVEGGRRRGPWGRRGKVSISRSVSIERIVTPGVYTKISRSSSVSVEGGRR
RGPWGYGRGLGGLSGSGDLDGLGGLGGLGGLGGRRGPWGRGYGSSGSASVSASVEGGRRRGPWG--------------------------------------
---------------------------------------RGYGSSGSASVSVSVEGGRR-------------------------------------------
RGPWG----------------------------------RGYGSSGSLSVSVSIERGIRRGPWGRRGKVSISRSVSIERIVTPGVYTKISRSSSVSVEGGRR
RGPWGYGRGLGGLSGSGDLDGLGGLGGLGGLGGRRGPWGRGYGSSGSASVSVSVEGGRRRGPWG--------------------------------------
---------------------------------------RGYGSSGSVSVSVSIERGIRRGPWGRRGKVSISRSVSIERIVTPGVYTKISRSSSVSVEGGRR
RGPWGYGRGLGGLSGSGDLDGLGGVGGLGGL-----------------------------------------------------------------------
----------------------------GGLGGRRGPWGRGYGSSGSASVSVSVEGGRRRGPWGR-------------------------------------
----------------------------------------GYGSSGSVSVSVSIERGIRRGPWGRRGKVSISRSVSIERIVTPGVYTKISRSSSVSVEGGRR
RGPWGYGRGLGGLSGSGDLDGLGGVGGLGGL-----------------------------------------------------------------------
----------------------------GGLGGRRGPWVRGYGSSGSASVSVSVEGGRRRGPWG--------------------------------------
---------------------------------------RGYGSSGSVSVSVSIERGIRRGPWGRRGKVSISRSVSIERIVTPGVYTKISRSSSVSVEGGRR
RGPWG----------------------------------RGYGSSGSVSVSVSIERGIRRGPWGRRGKVSISRSVSIERIVTPGVYTKISRSSSVSVEGGRR
RGPWG----------------------------------RGYGSSGSVSVSVSIERGIRRGPWGRRGKVSISRSVSIERIVTPGSYSKISRSSSVSVEGGIR
```

```
RGPWDRFGGYLSSRSSSIESVRPGRILAGLGRVGALGRSGVVPVPSVYTSHPGNSVKSPCKLSDFNLLVKVGNVRKSNGNC
```

Figure S2. Comparison of 5' ends (a) and 3' ends (b) of *L. lunatus* (L.lun) and *L. flavicornis* (L.fla) FibH with known FibH sequences from other caddisfly species. Abbreviations of the used species and source of the sequence: H.sp. - *Hesperophylax* sp. (Ashton et al., 2013); R.obl - *Rhyacophila obliterata* (GenBank: AB354587, AB354589); P.con - *Plectrocnemia conspersa* (GenBank: OL589410); S.tie - *Stenopsyche tienmushanensis* (Luo et al., 2018); S.mar - *Stenopsyche marmorata* (Wang et al., 2010); H.ang - *Hydropsyche angustipennis* (GenBank: AB214506, AB214507); P.els - *Parapsyche elsis* (Frandsen et al. 2019).

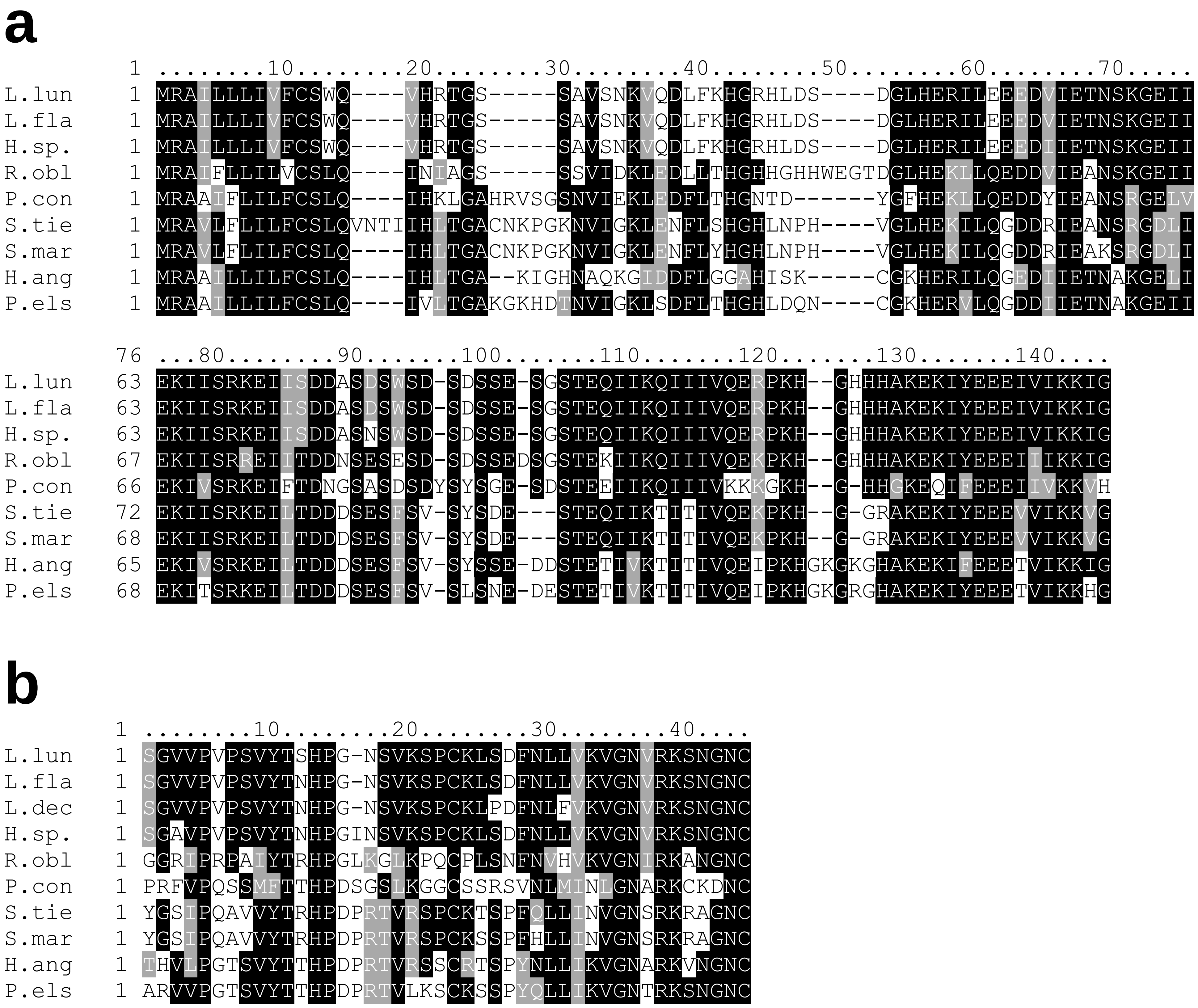

Figure S3. *L. lunatus* Cazs contain Ser- and Thr-rich motifs that frequently occur in adhesive proteins. a – comparison of *L. lunatus* Caz7 with *Stenopsyche angustata* Ser/Gly-rich protein (sequence ID S.ang7.801.2 in Wang et al. 2023) and *Galleria mellonella* sericin MG6 (GenBank: MG770319); b – comparison of *L. lunatus* Caz1 with *Andraca theae* mucin-like protein (GenBank: OQ418035) and *Drosophila melanogaster* glue protein Sgs3 (GenBank: NM_079300).

a

```
                  1 .........10........20........30........40........50........60
L.lun-Caz7        1 NKNAASSSSAASSASSAANKSSSSSSAASSASSAANKSSSSSSAASSAS-----------
S.ang7.801.2      1 AGSSSSSGGGSGASSSAASSSSSSSSSGGKSTSWSSTTDSSGSTGISASKCEDGKRPETY
G.mel-MG6         1 SNNSNSSNNSSSNSNSDSNSSSNSSSANNSNSDNSSSNSSSGSNSSSNS-----------

                 61 .........70........80........90.......100.......110.......120
L.lun-Caz7       50 ----------SAANKGSSSSSAASNASSAA---------NKGSSSSSAASSASNAAKNNA
S.ang7.801.2     61 SYTAGESASDSSSSSSSSSAASASDDEDAAMSRRSTKKCNQGEAKSFSWSSTTDSSGSTS
G.mel-MG6        50 -----SSSNNSSSNNSSSSENSSSNSSSGS---------NSGSSSSSNNSSSNSSSGSNS

                121 .......130.......140.......150.......160.......170.......180
L.lun-Caz7       91 SSSSSAAS------------SSSAAKKAACSGNKNAASSSSAAS----------------
S.ang7.801.2    121 FSSSKSED---GKAPVTVTYTTGDGKAAACAKTDDSTASSKSESNGYDDPTTTSTATGKG
G.mel-MG6        96 SSNSSSSNIVVSNNSSSSDNSSSNSSASQCSSSSSNNSSSNSSS----------------

                181 .......190.......200.......210.......220.......230.......240
L.lun-Caz7      123 ----------------------------------SASSAANKGSSSSSAASNASSASNKS
S.ang7.801.2    178 VAESVAINRCGKVSQSIERGNKSKGKRDCDGKSPNSDERGNRNNNNSNNDNRNNNGNSRS
G.mel-MG6       140 ----------------------------------GSNSSSNSSSSNNSNSNNNSSSNNSS

                241 .......250.......260.......270.......280.......290.......300
L.lun-Caz7      149 SSSSSAASSASSSANKNS-------------------------------------STSSA
S.ang7.801.2    238 SSSSSSSSSSSSSSNDNSNDGKGESFSWSSSTDDKGSTSISTSKSTDGGAPVTYTYTTGD
G.mel-MG6       166 SNSSSGSNSSSSSSSENSNSNSSSGSNSGSSSSSNNSSNVSNSGSSSSSNNSSSNSSSNS

                301 .......310.......320.......330.......340.......350.......360
L.lun-Caz7      172 ASSASSAANKNSSS----------------------------------------------
S.ang7.801.2    298 GKASASAKSDDSTSLSKSSSNGDNDPTTVADATGSGVAESVAVNRIGRSSHSFDGPEPRT
G.mel-MG6       226 NSGSSSSSNNNSSS----------------------------------------------

                361 .......370.......380.......390.......400...
L.lun-Caz7      186 ------------------SSAASSASSAANKNSSSSSAASSAS
S.ang7.801.2    358 EKSDGFERTAKPCSTGDGGSSSSSSSSSSNNSDGKSFSYSTST
G.mel-MG6       240 ------------------NNSSSSNNSSSNSSSGSNSGSSSSS
```

b

```
                  1 .........10........20........30........40........50........60
L.lun-Caz1        1 TRCPRTTCTTTCTTKCPKKTKCTTTKCTTTKCPKKTKCTTTKCPTTCTTKCPKKTKCTTT
A.the-MucL        1 GWVDRTIIGRKCK---PTPKPCTTTTCTPT-------------PKHCTKKC---------
D.mel-Sgs3        1 TTPKQTTTQLPCTT--PTTTKATTTKPTTTKA---TTTKATTTKPTTTKQTTTQLPCTTP

                 61 .........70........80........90.......100.......110.......120
L.lun-Caz1       61 RCTRTTRCPPTCTT-TRCTRTTRCPTTKCPKKTK-CTTTKCTTTKCPKKTKCTTTKCPTT
A.the-MucL       36 -----TTTPKPCTT-TTCTPTPKHCTTKCTTTPKPCTTTTCTPT-------------PKH
D.mel-Sgs3       56 TTTKQTTTQLPCTTPTTTKPTTTKPTTTKPTTTKPTTTKPTTTK--PTTTKPTTTKPTTT

                121 .......130.......140.......150.......160.......170.......180
L.lun-Caz1      119 CTTKC-PKKTKCTTTRCTTTKCPPTCRTKKTKCTTTKCPTTCTTKCPKKTKCTTTKCPTT
A.the-MucL       77 CTTTCTPTPKHCTTT------CTPT-------------PKHCTKKCSTTVK--------P
D.mel-Sgs3      114 KPTTTKPTTTKPTTTKPTTTKPTTTKPTTTKPTTTKPTTTKPTTTKPTTTKPTTTK---P

                181 .......190.......200.......210.
L.lun-Caz1      178 CTTKCPKKTKCTTTKCSTTTCKPTCSTTT-C
A.the-MucL      110 CTTKCTKKPPVTRTPWWPHT-KPTPPPTR-C
D.mel-Sgs3      171 TTTKPTTTKPTTPKPCGCKSCGPGGEPCNGC
```

Figure S4. Dendrogram depicting relationships between gene sequences coded by several clusters in separate loci of the *L. lunatus* genome. Both trees were constructed by Maximum likelihood method with the best model selected by ModelFinder stated in parentheses. a – Zonadhesin-like proteins (DCMut+F+G4); b – Pancreatic lipase-like proteins. (WAG+G4). Amino acid sequences were used for both trees, and the number of bootstrap replicates was 1000.

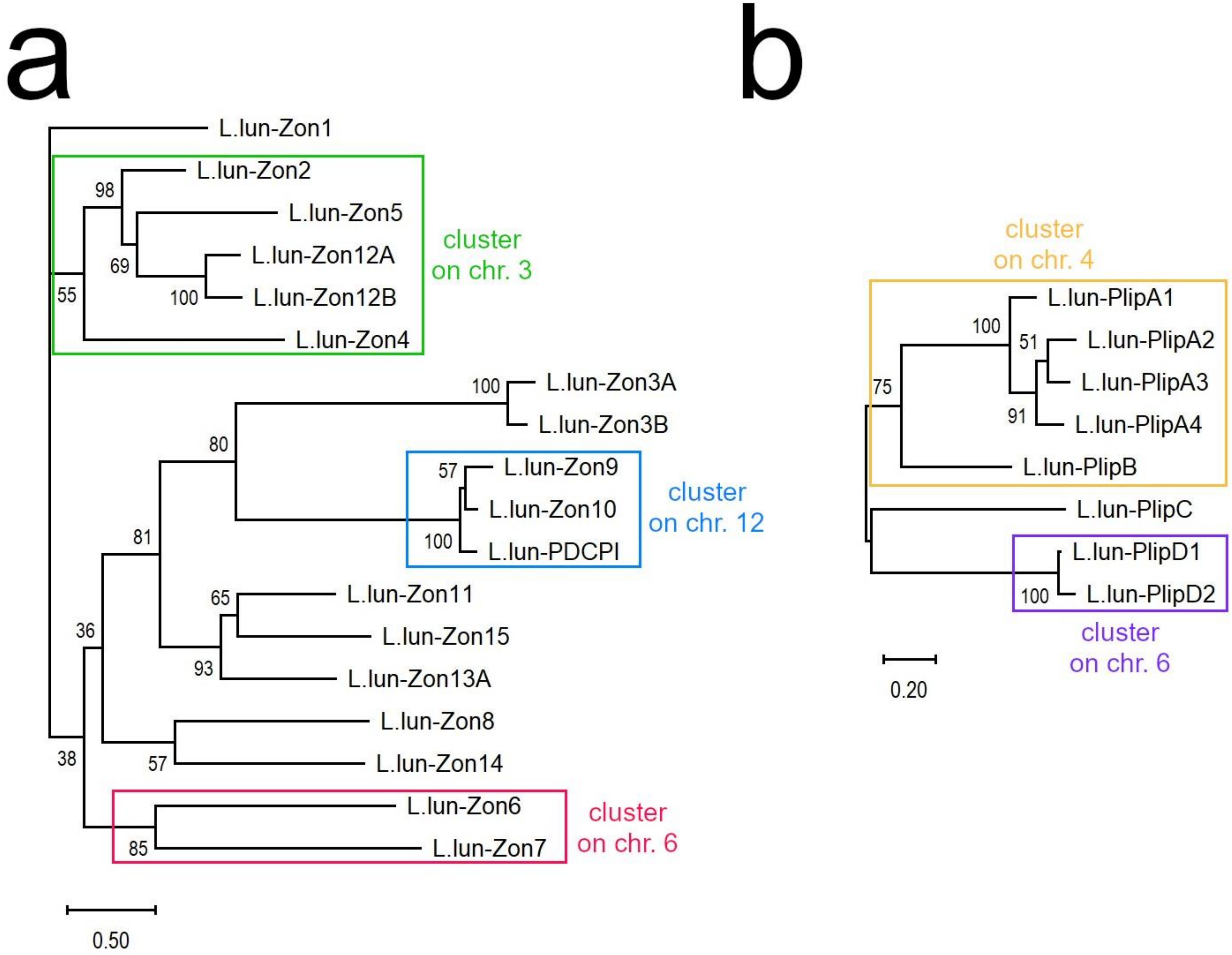

Table S1: Proteomic analysis of *L. lunatus*

Protein name	Symbol	GenBank accession	Signal peptide	Peptides	Razor + unique peptides	Unique peptides	Sequence coverage [%]	Unique + razor sequence coverage [%]	Unique sequence coverage [%]	Mol. weight [kDa]	Q-value	Score	Intensity	MS/MS count
Fibroin heavy chain	L.lun-FibH	BK063451	Yes	32	32	32	67.7	67.7	67.7	998.8	0	323.31	1783000000	47
Tubulin alpha	L.lun-TubA	OP700208	No	16	16	16	56.7	56.7	56.7	49.922	0	224.06	33226000	34
Zonadhesin-like protein 2	L.lun-Zon2	BK062787	Yes	18	18	16	35.4	35.4	32.8	50.886	0	323.31	25181000	28
Elongation factor 1 alpha	L.lun-EF1A	OP700209	No	8	8	8	24.7	24.7	24.7	50.71	0	74.499	22689000	26
Fibroin light chain	L.lun-FibL	BK062791	Yes	6	6	6	43	43	43	26.064	0	31.571	19384000	9
Actin	L.lun-Act	OP700210	No	14	14	14	54.8	54.8	54.8	41.821	0	165.43	11217000	23
Vacuolar protein sorting-associated protein 13C	L.lun-VPS13C	OP700211	No	2	2	2	1	1	1	234.88	0	11.576	9412700	3
Peroxinectin 1 - transcription variant X1/X2	L.lun-Pxn1_X1/X2	BK062798	Yes	12	12	9	24	24	19.4	76.048	0	145.72	7887600	21
Tubulin beta 1	L.lun-TubB1	OP700212	No	14	14	7	47.9	47.9	30.2	50.217	0	92.845	5597500	23
Zonadhesin-like protein 4	L.lun-Zon4	BK062787	Yes	8	8	8	20.5	20.5	20.5	75.417	0	138.71	5428900	9
Zonadhesin-like protein 3B	L.lun-Zon3B	BK062809	Yes	3	3	3	21.2	21.2	21.2	18.718	0	63.742	4502500	4
Zonadhesin-like protein 6	L.lun-Zon6	BK062793	Yes	9	9	9	29	29	29	30.319	0	144.07	4088800	10
Small multicopy peptide A7/A8	L.lun-SMPA7/A8	BK062800	Yes	1	1	1	26.5	26.5	26.5	5.7145	0	17.9	3856600	2
Small multicopy peptide A1/A2	L.lun-SMPA1/A2	BK062800	Yes	1	1	1	13.7	13.7	13.7	6.0789	0	9.3832	3702800	1
Zonadhesin-like protein 1	L.lun-Zon1	BK062784	Yes	13	13	13	22.9	22.9	22.9	107.04	0	94.217	3639100	14
Zonadhesin-like protein 3A	L.lun-Zon3A	BK062786	Yes	4	4	4	38	38	38	18.091	0	27.76	3368500	6
Small YEC-rich multicopy peptide 3/4	L.lun-YEC3/4	BK062782	Yes	2	2	2	35	35	35	9.1433	0	19.503	3321700	2
DDB1- and CUL4-associated factor 8	L.lun-DCAF8	OP700213	No	1	1	1	1.3	1.3	1.3	83.162	0	2.7191	3116000	1
Glycerol-3-phosphate dehydrogenase	L.lun-G3PD	OP700214	No	1	1	1	6	6	6	38.203	0	6.6197	3105000	2
Small multicopy peptide A5	L.lun-SMPA5	BK062800	Yes	2	2	2	37.3	37.3	37.3	6.0989	0	25.619	2752800	6
Small multicopy peptide B1/B2	L.lun-SMPB1/B2	BK062800	Yes	2	2	2	30.5	30.5	30.5	6.4179	0	10.842	2283600	2
AT rich 24kDa protein B	L.lun-AT24B	BK062780	Yes	6	6	3	31.2	31.2	18.2	26.466	0	89.77	2075600	8
Pancreatic lipase-related protein A3	L.lun-PlipA3	BK062789	Yes	6	6	6	21.9	21.9	21.9	37.582	0	88.879	1959400	6
Uncharacterized conserved CG3556-like 1	L.lun-UchC1	BK062781	Yes	5	5	5	37.3	37.3	37.3	16.941	0	29.776	1680300	6
Arginine kinase	L.lun-ArgK	OP700215	No	3	3	3	10.7	10.7	10.7	39.88	0	24.745	1410500	6
Zonadhesin-like protein 8	L.lun-Zon8	BK062812	Yes	7	7	7	17.6	17.6	17.6	55.594	0	46.734	1390800	9
Biorientation of chromosomes in cell division protein 1-like 1	L.lun-BCCD1	OP700216	No	2	2	2	1.5	1.5	1.5	152.95	0.006494	0.34953	1378900	1
Acetyl-CoA carboxylase	L.lun-ACC	OP700217	No	6	6	6	4.3	4.3	4.3	258.73	0	15.466	1315900	6
Peroxinectin 2	L.lun-Pxn2	BK062798	Yes	5	2	2	7.6	3.5	3.5	84.438	0	17.536	1314000	3
Cadhesin 3A/3B	L.lun-Caz3A/3B	BK062803	Yes	4	4	4	12.4	12.4	12.4	42.053	0	40.599	1301300	5
Esterase 3	L.lun-Est3	BK062795	Yes	7	7	6	15.8	15.8	14.6	62.316	0	20.494	1241700	7
LA rich 27kDa protein	L.lun-LA27	BK062792	Yes	3	3	3	14.1	14.1	14.1	30.004	0	23.799	1224000	5
Polyubiquitin	L.lun-Puq	OP700218	No	4	4	4	57.5	57.5	57.5	85.699	0	58.352	1184100	4
LS rich 29kDa protein	L.lun-LS29	BK062792	Yes	3	3	3	12.1	12.1	12.1	31.712	0	10.481	1116300	3
ATP synthase subunit beta, mitochondrial	L.lun-ATPB	OP700219	No	5	5	5	17.4	17.4	17.4	55.112	0	8.8976	1085000	4
Histone H4	L.lun-His4	OP700220	No	5	5	5	50.5	50.5	50.5	11.381	0	39.843	1076500	5
Ribosomal protein P1	L.lun-RpP1	OP700221	No	1	1	1	20.7	20.7	20.7	11.168	0	11.628	1057900	3
Guanine nucleotide-binding protein subunit beta-like	L.lun-GNBPB	OP700222	No	2	2	2	8.8	8.8	8.8	35.5	0	30.566	1040000	2
Esterase 1	L.lun-Est1	BK062795	Yes	5	4	3	8.6	7.4	5.6	61.726	0	13.796	1016200	4
Ribosomal protein L11	L.lun-RpL11	OP700223	No	4	4	4	23.7	23.7	23.7	22.036	0	26.302	943030	5
Esterase 2	L.lun-Est2	BK062795	Yes	5	3	3	10.6	7.6	7.6	61.647	0	11.746	936790	3
Spectrin beta chain, non-erythrocytic	L.lun-SpcrB	OP700224	No	4	4	4	1	1	1	482.01	0	4.0661	923330	1
Small multicopy peptide A4/A6	L.lun-SMPA4/A6	BK062800	Yes	2	2	2	40.7	40.7	40.7	6.4913	0	6.2015	918650	3
Histone H2A variant	L.lun-His2A.V	OP700225	No	2	2	2	12.4	12.4	12.4	13.443	0	12.025	915490	2
Clathrin heavy chain	L.lun-Chc	OP700226	No	4	4	4	3.6	3.6	3.6	190.66	0	55.396	902820	4
Enhancer of mRNA-decapping protein 4	L.lun-EMD4	OP700227	No	2	2	2	1.6	1.6	1.6	146.73	0	1.9585	887100	1
Translation elongation factor 2	L.lun-TEF2	OP700228	No	2	2	2	5.5	5.5	5.5	94.73	0	4.2106	846610	2
ADP-ribosylation factor 1-like	L.lun-ARF1	OP700229	No	5	5	5	50.5	50.5	50.5	20.736	0	43.504	787180	8
Zonadhesin-like protein 9/10	L.lun-Zon9/10	BK062805	Yes	4	4	4	9.6	9.6	9.6	50.002	0	19.962	781850	3
UTP-glucose-1-phosphate uridylyltransferase	L.lun-UG1PU	OP700230	No	1	1	1	4.5	4.5	4.5	57.231	0	1.4366	775490	1
Myosin heavy chain, non-muscle	L.lun-MyoNM	OP700231	No	2	2	2	1.7	1.7	1.7	226.33	0	3.549	715290	1
S-adenosylmethionine synthetase	L.lun-SAMS	OP700232	No	2	2	2	5.2	5.2	5.2	44.441	0	11.533	634670	2
Ribosomal protein S15	L.lun-RpS15	OP700233	No	2	2	2	20.4	20.4	20.4	17.078	0	2.2783	617130	3
PEVK-like protein	L.lun-PEVK	BK062794	Yes	5	5	5	14.6	14.6	14.6	58.954	0	7.0697	597090	6
Pancreatic lipase-related protein C	L.lun-PlipC	BK062788	Yes	6	6	6	17.8	17.8	17.8	41.739	0	16.803	563530	6
ADP,ATP carrier protein	L.lun-AACP	OP700234	No	4	4	4	17.3	17.3	17.3	32.821	0	4.5281	541280	4
C-rich 30kDa protein A/B	L.lun-C30A/B	BK062797	Yes	4	4	4	19.3	19.3	19.3	32.21	0	16.954	512460	6
Glyceraldehyde-3-phosphate dehydrogenase	L.lun-Gdh3PD	OP700235	No	1	1	1	2.1	2.1	2.1	35.205	0	7.4771	482340	2
LAN32 homolog	L.lun-LAN32	BK062792	Yes	4	4	4	14.2	14.2	14.2	34.185	0	79.769	468500	3
Serine protease 2	L.lun-SP2	BK062802	Yes	2	2	2	6.9	6.9	6.9	43.563	0	21.015	442490	2
Cadhesin 6	L.lun-Caz6	BK062803	Yes	4	4	4	18.8	18.8	18.8	38.464	0	21.46	426120	3
Heat shock protein 70-like	L.lun-Hsp70	OP700236	No	4	4	1	7.8	7.8	1.5	71.728	0	28.696	411810	4
Apolipoprotein D	L.lun-AlpD	BK062810	Yes	2	2	2	14.9	14.9	14.9	21.494	0	2.3373	409170	1
26S protease regulatory subunit 6A-B	L.lun-26SPR6A-B	OP700237	No	2	2	2	6.5	6.5	6.5	47.688	0	4.6729	407000	3
Glycogen phosphorylase	L.lun-Glyp	OP700238	No	3	3	3	3.2	3.2	3.2	97.188	0	45.047	405680	3
Thioredoxin domain-containing protein 12-like A/B	L.lun-Trx12A/B	BK062808	Yes	2	2	2	12.3	12.3	12.3	17.531	0	7.4358	404340	2
Histone H3	L.lun-His3	OP700239	No	2	2	2	11.8	11.8	11.8	15.328	0	7.6271	394660	2
Ribosomal protein S3	L.lun-RpS3	OP700240	No	1	1	1	5.4	5.4	5.4	26.436	0	17.751	375450	1
Zonadhesin-like protein 11	L.lun-Zon11	BK062808	Yes	3	3	3	8.5	8.5	8.5	55.157	0	10.043	353670	2
KD-rich 15kDa protein	L.lun-KD15	BK062801	Yes	1	1	1	8.5	8.5	8.5	17.448	0	4.2769	349900	2
Eukaryotic peptide chain release factor GTP-binding subunit ERF3A	L.lun-ERF3A	OP700241	No	2	2	2	6.7	6.7	6.7	64.545	0	6.0911	338120	3
Calmodulin	L.lun-Clm	OP700242	No	2	2	2	23.5	23.5	23.5	16.81	0	11.391	330700	3
Tubulin beta 2	L.lun-TubB2	OP700243	No	9	2	2	23.8	6.1	6.1	50.076	0	10.62	316350	4
Zonadhesin-like protein 12A	L.lun-Zon12A	BK062787	Yes	3	2	2	14.6	11.2	11.2	28.699	0	3.4951	312970	2
Heat shock protein 83-like	L.lun-Hsp83	OP700244	No	2	2	2	3.5	3.5	3.5	81.921	0	9.699	301820	2
26S protease regulatory subunit 4	L.lun-26SPR4	OP700245	No	1	1	1	4.8	4.8	4.8	49.253	0	3.7892	281480	1
26S protease regulatory subunit 7	L.lun-26SPR7	OP700246	No	1	1	1	3.7	3.7	3.7	48.597	0	7.8162	261660	1
T-complex protein 1 subunit beta	L.lun-TC1B	OP700247	No	1	1	1	2.8	2.8	2.8	57.451	0	46.925	257150	2
RING finger protein 17-like	L.lun-RING17	OP700248	No	1	1	1	0.5	0.5	0.5	158.72	0	6.7752	252930	1
Cadhesin 11	L.lun-Caz11	BK062804	Yes	2	2	2	3	3	3	127.58	0	5.5669	245240	3
Sericotropin-like protein 1/2	L.lun-Srctr1/2	BK062806	Yes	2	2	2	14.7	14.7	14.7	14.983	0	3.766	243400	2
Endoplasmic reticulum chaperone BiP	L.lun-ERBiP	OP700249	No	4	2	2	8.8	4.6	4.6	72.991	0	9.9481	232440	2
Glutamine--fructose-6-phosphate aminotransferase [isomerizing] 1	L.lun-GF6PA	OP700250	No	1	1	1	1.9	1.9	1.9	75.538	0	14.065	184300	1
Zonadhesin-like protein 12B	L.lun-Zon12B	BK062787	Yes	3	1	0	5.5	2.3	0	42.232	0	2.8466	183870	1
Cilia- and flagella-associated protein 45	L.lun-CFA45	OP700251	No	1	1	1	1.9	1.9	1.9	62.113	0	8.4212	183860	0
T-complex protein 1 subunit eta	L.lun-TC1E	OP700252	No	2	2	2	4.8	4.8	4.8	58.69	0	8.8343	169720	1
Pyruvate carboxylase, mitochondrial	L.lun-Pcx	OP700253	No	1	1	1	1.8	1.8	1.8	132.34	0	1.221	164760	1
Myb-binding protein 1A-like	L.lun-MybB1A	OP700254	No	2	2	2	1.6	1.6	1.6	145.6	0	1.1123	153940	0
TSAG-rich 18kDa protein	L.lun-TSAG18	BK062803	Yes	1	1	1	17.2	17.2	17.2	19.958	0.006289	0.24228	151940	1
26S proteasome non-ATPase regulatory subunit 11	L.lun-26SPR11	OP700255	No	2	2	2	5.5	5.5	5.5	47.226	0	8.9218	133720	1
Aspartate--tRNA ligase, cytoplasmic	L.lun-AspTL	OP700256	No	1	1	1	3.6	3.6	3.6	59.734	0	10.968	132100	1
Phosphoribosyl pyrophosphate synthetase	L.lun-PrPpS	OP700257	No	1	1	1	4.3	4.3	4.3	37.948	0	9.3156	127720	1
GTP-binding protein 128up	L.lun-GB128	OP700258	No	1	1	1	3.8	3.8	3.8	40.933	0	5.1018	112250	1
26S proteasome non-ATPase regulatory subunit 1	L.lun-26SPR1	OP700259	No	1	1	1	1.5	1.5	1.5	110.21	0	4.8003	108870	1
Heat shock protein 70-like A	L.lun-Hsp70A	OP700260	No	3	1	1	6.3	2.5	2.5	68.875	0	4.4419	105820	1
Ribosomal protein L17	L.lun-RpL17	OP700261	No	3	3	2	10.2	10.2	5.9	21.543	0	16.077	102480	2
Zonadhesin-like protein 13A	L.lun-Zon13A	BK062785	Yes	2	2	2	4.5	4.5	4.5	55.193	0	5.3003	92852	2
Triosephosphate isomerase	L.lun-Tpi	OP700262	No	1	1	1	4.8	4.8	4.8	26.27	0	3.1265	91581	1
Ribosomal protein P2	L.lun-RpL2	OP700263	No	1	1	1	11.8	11.8	11.8	11.366	0	21.259	84364	1
26S protease regulatory subunit 6B	L.lun-26SPR6B	OP700264	No	1	1	1	4.6	4.6	4.6	47.059	0	2.3012	68605	1
Heat shock protein 70-like B	L.lun-Hsp70B	OP700265	No	2	1	1	3.7	1.9	1.9	69.336	0	4.517	60393	1
Cadhesin 9	L.lun-Caz9	BK062803	Yes	1	1	1	1.6	1.6	1.6	97.329	0	5.2571	57709	1
cAMP-dependent protein kinase type I regulatory subunit	L.lun-CDKIR	OP700266	No	1	1	1	3.5	3.5	3.5	41.771	0	2.6104	54179	1
Cadhesin 4	L.lun-Caz4	BK062803	Yes	1	1	1	1.1	1.1	1.1	87.209	0.006849	0.57286	52718	1
Vesicle-fusing ATPase 1-like	L.lun-VFA1	OP700267	No	1	1	1	2.4	2.4	2.4	82.199	0	4.9057	0	1
Tropomyosin	L.lun-Tmyo	OP700268	No	1	1	1	3.2	3.2	3.2	32.662	0	18.501	0	1
Ribosomal protein S15A	L.lun-RpS15A	OP700269	No	1	1	1	10.8	10.8	10.8	14.827	0	10.136	0	1
Cadhesin 12	L.lun-Caz12	BK062804	Yes	1	1	1	4.7	4.7	4.7	34.351	0	1.5815	0	1
Glutaminyl-peptide cyclotransferase	L.lun-GPC	OP700270	No	1	1	1	2.9	2.9	2.9	39.225	0	4.529	0	1
Glycogen [starch] synthase	L.lun-GcgS	OP700271	No	1	1	1	1.6	1.6	1.6	79.637	0	1.927	0	1
Eukaryotic translation initiation factor 4A	L.lun-ETIF4A	OP700272	No	1	1	1	2.4	2.4	2.4	48.218	0	5.3012	0	0
Exocyst complex component 8	L.lun-ECC8	OP700273	No	1	1	1	1.2	1.2	1.2	78.47	0.006369	0.2997	0	0
Cytosolic 10-formyltetrahydrofolate dehydrogenase	L.lun-C10FH	OP700274	No	1	1	1	1.7	1.7	1.7	102.42	0.006623	0.37165	0	0
Cadhesin 1	L.lun-Caz1	BK062803	Yes	1	1	1	2.7	2.7	2.7	36.225	0.006024	0.21416	0	0
Cadhesin 10	L.lun-Caz10	BK062804	Yes	2	2	2	1.3	1.3	1.3	224.67	0.00641	0.31112	0	1
Kielin/chordin-like protein	L.lun-KCHP	OP700275	No	2	2	2	1.6	1.6	1.6	157.09	0	2.1831	0	2

Table S2: Proteomic analysis of *L. flavicornis*

Protein name	Symbol	GenBank accession	Signal peptide	Peptides	Razor + unique peptides	Unique peptides	Sequence coverage [%]	Unique + razor sequence coverage [%]	Unique sequence coverage [%]	Mol. weight [kDa]	Q-value	Score	Intensity	MS/MS count
Fibroin heavy chain	L.fla-FibH	OP700089	Yes	41	41	41	50.6	50.6	50.6	124.57	0	323.31	6918300000	128
Zonadhesin-like protein 3A	L.fla-Zon3A	OP700090	Yes	10	10	10	60.1	60.1	60.1	18.099	0	79.208	2418400000	33
Zonadhesin-like protein 2A	L.fla-Zon2A	OP700091	Yes	36	36	8	70.6	70.6	13.6	51.851	0	323.31	1734100000	58
Small multicopy peptide A1	L.fla-SMPA1	OP700092	Yes	2	2	2	32.7	32.7	32.7	5.7606	0	9.3125	380560000	2
Fibroin light chain	L.fla-FibL	OP700093	Yes	4	4	4	18.1	18.1	18.1	25.979	0	81.107	378390000	21
Zonadhesin-like protein 3B	L.fla-Zon3B	OP700094	Yes	6	6	6	35.7	35.7	35.7	18.693	0	149.82	348180000	11
Zonadhesin-like protein 6	L.fla-Zon6	OP700095	Yes	10	10	10	52.5	52.5	52.5	30.278	0	180.14	325050000	20
Peroxinectin 1 - transcription variant X1/X2	L.fla-Pxn1_X1/X2	OP700096/OP700097	Yes	20	20	20	41.3	41.3	41.3	75.79	0	323.31	318850000	43
Hexamerin 1	L.fla-Hex1	OP700098	Yes	20	20	20	32.2	32.2	32.2	82.406	0	228.64	291810000	38
Zonadhesin-like protein 3C	L.fla-Zon3C	OP700099	Yes	6	6	6	63.1	63.1	63.1	18.713	0	105.2	191330000	9
Zonadhesin-like protein 1A	L.fla-Zon1A	OP700100	Yes	31	31	31	49.2	49.2	49.2	113.38	0	323.31	153730000	41
Actin	L.fla-Act	OP700101	No	14	14	14	44.4	44.4	44.4	41.821	0	90.312	70327000	24
Small multicopy peptide A2	L.fla-SMPA2	OP700102	Yes	2	1	1	37.3	37.3	37.3	6.0618	0	4.8933	63161000	2
Zonadhesin-like protein 4	L.fla-Zon4	OP700103	Yes	17	17	17	36.5	36.5	36.5	85.982	0	125.57	62685000	21
Zonadhesin-like protein 11A	L.fla-Zon11A	OP700104	Yes	24	24	14	45.1	45.1	26.2	88.472	0	152.2	57319000	33
Zonadhesin-like protein 8	L.fla-Zon8	OP700105	Yes	16	16	16	33.3	33.3	33.3	67.754	0	131.65	54126000	24
Zonadhesin-like protein 9	L.fla-Zon9	OP700106	Yes	18	18	6	49.3	49.3	22.7	50.274	0	117.17	46659000	23
Esterase 1	L.fla-Est1	OP700107	Yes	15	15	15	40.6	40.6	40.6	61.62	0	220.51	44694000	21
Small multicopy peptide A3	L.fla-SMPA3	OP700108	Yes	2	2	2	40.7	40.7	40.7	6.4542	0	16.982	42612000	5
Pancreatic lipase-like A3	L.fla-PlipA3	OP700109	Yes	8	8	8	30	30	30	37.256	0	162.54	35876000	8
Small YEC-rich multicopy peptide 4	L.fla-YEC4	OP700110	Yes	4	4	4	55	55	55	8.9471	0	67.353	35100000	5
Cadhesin 6A	L.fla-Caz6A	OP700111	Yes	8	8	4	24	24	16.8	38.934	0	38.809	34639000	13
Zonadhesin-like protein 12	L.fla-Zon12	OP700112	Yes	8	8	8	42.5	42.5	42.5	35.597	0	75.47	31360000	13
Elongation factor 1 alpha	L.fla-EF1A	OP700113	No	8	8	8	15.8	15.8	15.8	50.532	0	18.471	30813000	7
Zonadhesin-like protein 11B	L.fla-Zon11B	OP700114	Yes	19	9	9	48.3	23.8	23.8	67.759	0	69.704	30207000	10
Zonadhesin-like protein 13	L.fla-Zon13	OP700115	Yes	9	9	9	34	34	34	55.231	0	80.055	27330000	11
Histone H4	L.fla-His4	OP700116	No	6	6	6	51.5	51.5	51.5	11.381	0	33.848	26152000	8
LAN32 homolog	L.fla-LAN32	OP700117	Yes	7	7	7	29.7	29.7	29.7	33.982	0	25.024	25875000	8
Hexamerin 2	L.fla-Hex2	OP700118	Yes	14	14	14	16.1	16.1	16.1	92.107	0	89.286	24749000	16
LA27-like	L.fla-LA27	OP700119	Yes	9	9	9	21.7	21.7	21.7	30.581	0	35.493	22892000	10
TSAG18-like	L.fla-TSAG18	OP700120	Yes	1	1	1	6.7	6.7	6.7	20.758	0	25.325	18983000	1
ATP synthase subunit beta	L.fla-ATPB	OP700121	No	9	9	9	21.7	21.7	21.7	55.001	0	38.85	18031000	9
Glutaminyl-peptide cyclotransferase	L.fla-GPC	OP700122	No	9	9	9	32.8	32.8	32.8	39.245	0	86.437	14201000	10
ATP synthase subunit alpha	L.fla-ATPA	OP700123	No	8	8	8	15.6	15.6	15.6	59.323	0	29.046	14091000	8
Pancreatic lipase-like C	L.fla-PlipC	OP700124	Missing	5	5	5	27.5	27.5	27.5	31.326	0	19.946	13908000	5
Histone H2A.V	L.fla-His2A.V	OP700125	No	3	3	1	20.2	20.2	7.8	13.443	0	9.5727	12573000	3
Cadhesin 12	L.fla-Caz12	OP700126	Yes	3	3	3	13.8	13.8	13.8	35.666	0	57.116	12493000	3
Heat shock protein 70-like	L.fla-Hsp70	OP700127	No	7	7	4	13.6	13.6	7.5	71.698	0	32.666	10711000	7
C-rich 30-kDa protein	L.fla-C30	OP700128	Yes	5	5	5	24.7	24.7	24.7	32.341	0	67.124	10017000	8
Small multicopy peptide A5	L.fla-SMPA5	OP700129	Yes	2	2	2	40.7	40.7	40.7	6.4582	0	9.422	9924600	8
Tubulin alpha	L.fla-TubA	OP700130	No	4	4	4	10.7	10.7	10.7	49.922	0	18.956	9629600	5
Pancreatic lipase-like D	L.fla-PlipD	OP700131	Yes	3	3	3	11.8	11.8	11.8	36.86	0	44.127	7402000	3
Polyubiquitin - partial/Ubiquitin C-terminus	L.fla-Puq	OP700132	No	3	3	3	28	28	28	23.718	0	42.883	6500400	4
Small multicopy peptide B	L.fla-SMPB	OP700133	Yes	2	2	2	25	25	25	6.389	0	5.4559	6430700	2
LS29-like	L.fla-LS29	OP700134	Yes	3	3	3	16.2	16.2	16.2	31.477	0	51.78	6078000	3
Glyceraldehyde-3-phosphate dehydrogenase	L.fla-G3PD	OP700135	No	5	5	5	16.9	16.9	16.9	35.221	0	18.925	6072900	8
PEVK-like protein	L.fla-PEVK	OP700136	Yes	8	8	8	26	26	26	60.267	0	32.225	5836000	11
Adenosine deaminase 2-like	L.fla-ADA2	OP700137	Yes	9	9	9	22.8	22.8	22.8	57.518	0	49.139	5667100	10
KD rich 15kDa protein	L.fla-KD15	OP700138	Yes	3	3	3	19	19	19	17.301	0	15.073	5510500	4
Cadhesin 11	L.fla-Caz11	OP700139	Yes	4	4	4	5.8	5.8	5.8	131.63	0	10.858	5345600	5
Ferritin 1	L.fla-Fer1	OP700140	Yes	4	4	4	8.7	8.7	8.7	26.147	0	12.357	5095200	5
Serine protease 2	L.fla-SP2	OP700141	Yes	5	5	5	19.9	19.9	19.9	43.373	0	120	4879700	5
Tubulin beta 1	L.fla-TubB1	OP700142	No	9	9	4	28.4	28.4	15	50.217	0	100.19	4585400	11
Uncharacterized conserved CG3556-like 1	L.fla-UchC1	OP700143	Yes	3	3	3	22.1	22.1	22.1	16.929	0	9.2665	4426700	2
Serpin	L.fla-Srp	OP700144	Yes	5	5	5	15.6	15.6	15.6	44.756	0	50.052	4179700	4
Histone H2B	L.fla-His2B	OP700145	No	4	4	4	35.5	35.5	35.5	13.796	0	34.592	4039700	6
Fructose 1,6-bisphosphate aldolase	L.fla-FBPAP	OP700146	No	4	4	4	14	14	14	39.419	0	19.204	3759100	4
Zonadhesin-like protein 14	L.fla-Zon14	OP700147	Yes	5	5	5	33.3	33.3	33.3	36.2	0	24.087	3692500	5
Lysozyme	L.fla-Lzm	OP700148	Yes	2	2	2	18.6	18.6	18.6	15.271	0	3.5989	3124000	2
Heat shock protein 83-like	L.fla-Hsp83	OP700149	No	2	2	2	2.4	2.4	2.4	81.921	0	4.4497	2675200	1
Elongation factor Tu	L.fla-EFTu	OP700150	No	1	1	1	1.9	1.9	1.9	51.378	0	2.639	2577400	1
Cadhesin 9	L.fla-Caz9	OP700151	Yes	2	2	2	5.3	5.3	5.3	81.662	0	129.22	2573800	4
Cadhesin 8	L.fla-Caz8	OP700152	Yes	1	1	1	1.6	1.6	1.6	103.77	0	114.07	2509200	1
Protein lava lamp	L.fla-Lva	OP700153	No	6	6	6	2.7	2.7	2.7	365.94	0	5.7056	2451600	2
Zonadhesin-like protein 10	L.fla-Zon10	OP700154	Yes	14	2	2	24.9	5.5	5.5	68.903	0	3.0388	2391500	1
AT rich 24kDa protein	L.fla-AT24	OP700155	Yes	4	4	4	22.1	22.1	22.1	26.301	0	18.894	2267300	6
14-3-3 protein zeta	L.fla-YWHAZ	OP700156	No	2	2	2	7.3	7.3	7.3	28.244	0	19.42	2257500	3
Tubulin beta 2	L.fla-TubB2	OP700157	No	7	2	2	19.5	6.1	6.1	50.076	0	45.966	2202500	3
Zonadhesin-like protein 1B	L.fla-Zon1B	OP700158	Yes	1	1	1	11.5	11.5	11.5	10.345	0	6.185	1943300	1
Small YEC-rich multicopy peptide 1	L.fla-YEC1	OP700159	Yes	3	3	3	56.4	56.4	56.4	8.76	0	19.106	1903900	3
Ferritin 2	L.fla-Fer2	OP700160	Yes	4	4	4	18.5	18.5	18.5	23.523	0	7.5646	1879800	3
Peptidylprolyl isomerase B	L.fla-PpiB	OP700161	Yes	4	4	4	17.9	17.9	17.9	22.66	0	6.5137	1839200	3
SUN domain-containing ossification factor-like	L.fla-SucoL	OP700162	No	4	4	4	3.4	3.4	3.4	132.08	0	3.5847	1647100	1
Transcription elongation factor SPT6	L.fla-Spt6	OP700163	No	2	2	2	2	2	2	202.79	0	2.403	1539300	1
Peroxiredoxin 1-like	L.fla-Prdx1	OP700164	No	1	1	1	5.6	5.6	5.6	21.822	0	6.3659	1513000	1
Small multicopy peptide A4	L.fla-SMPA4	OP700165	Yes	2	2	1	33.3	33.3	33.3	6.0759	0	30.525	1512700	2
Imaginal disc growth factor	L.fla-IDGF	OP700166	Yes	7	7	7	21.5	21.5	21.5	48.404	0	40.939	1419700	7
Odorant-binding protein 1	L.fla-OBP1	OP700167	Yes	3	3	3	17.6	17.6	17.6	14.711	0	6.8446	1400900	4
Tropomyosin	L.fla-Tmyo	OP700168	No	3	3	3	13.7	13.7	13.7	32.662	0	4.9253	1327900	3
Phenoloxidase subunit 1	L.fla-PPO1	OP700169	No	5	5	5	7.9	7.9	7.9	34.665	0	15.892	1253000	5
Voltage-dependent anion-selective channel	L.fla-VDAC	OP700170	No	1	1	1	5	5	5	30.384	0	1.9628	1087700	1
Heat shock protein 70-like A	L.fla-Hsp70A	OP700171	No	4	1	1	8.9	2.5	2.5	68.861	0	2.6692	980610	1
Cadhesin 3A/3B	L.fla-Caz3A/3B	unresolved paralogs	Yes	2	2	2	13.3	13.3	13.3	56.141	0	7.842	955390	2
Eukaryotic translation initiation factor 4A	L.fla-ETIF4A	OP700172	No	3	3	3	12	12	12	48.218	0	19.822	908320	3
Myrosinase 1-like	L.fla-Myr1	OP700173	Yes	1	1	1	1.4	1.4	1.4	59.156	0	3.0961	843320	1
Porphobilinogen deaminase	L.fla-PBGD	OP700174	No	1	1	1	2.3	2.3	2.3	68.686	0	3.1253	826950	0
Malate dehydrogenase, mitochondrial	L.fla-MDH	OP700175	No	2	2	2	7.3	7.3	7.3	35.654	0	12.474	784180	2
ADP,ATP carrier protein	L.fla-AACP	OP700176	No	2	2	2	6.6	6.6	6.6	32.893	0	4.8738	764440	2
Arginine kinase	L.fla-ArgK	OP700177	No	4	4	4	11.8	11.8	11.8	39.896	0	9.0236	732720	4
Zonadhesin-like protein 2B	L.fla-Zon2B	OP700178	Yes	29	1	1	61.2	4.1	4.1	51.934	0	6.2003	714880	2
Pyruvate dehydrogenase E1 component subunit alpha, mitochondrial	L.fla-PDHA1	OP700179	No	1	1	1	2.5	2.5	2.5	43.992	0	2.4729	714010	1
RING finger protein 17-like	L.fla-RING17	OP700180	No	2	2	2	1.8	1.8	1.8	159.04	0	4.3102	707420	1
C-type lectin domain containing 46kDa protein	L.fla-CTL46	OP700181	Yes	3	3	3	9.2	9.2	9.2	49.62	0	12.344	678620	3
Cadhesin 1	L.fla-Caz1	OP700182	Yes	2	2	2	5.3	5.3	5.3	45.369	0	3.3654	569110	1
Zonadhesin-like protein 15	L.fla-Zon15	OP700183	Yes	1	1	1	5.5	5.5	5.5	32.376	0	4.9542	554950	2
Odorant-binding protein 2	L.fla-OBP2	OP700184	Yes	3	3	3	18.3	18.3	18.3	21.095	0	34.115	524610	4
Zonadhesin-like protein 5	L.fla-Zon5	OP700185	Yes	4	2	2	14.7	11.5	11.5	39.987	0	5.5622	499770	3
Cadhesin 6B	L.fla-Caz6B	OP700186	Yes	5	1	1	12.9	5.2	5.2	36.075	0	18.059	438520	1
Prostatic acid phosphatase-like	L.fla-PAP	OP700187	Yes	2	2	2	5.4	5.4	5.4	42.568	0	11.398	432370	2
Antichymotrypsin-2-like	L.fla-Achtr2	OP700188	Yes	2	2	2	4.3	4.3	4.3	51.576	0	12.587	429110	2
Non-secretory mucin-like protein	L.fla-nsMuc	OP700189	No	1	1	1	0.4	0.4	0.4	290.29	0	2.9708	360540	1
Histone H2A	L.fla-His2A	OP700190	No	3	1	1	31.5	18.5	18.5	13.363	0	3.5222	317420	1
Peptidylprolyl isomerase-like	L.fla-PpiL	OP700191	No	1	1	1	4.9	4.9	4.9	22.283	0.006803	1.6012	301490	0
Superoxid dismutase 3	L.fla-SOD3	OP700192	Yes	2	2	2	15.9	15.9	15.9	16.857	0	4.1612	278530	2
Kynurenine formamidase	L.fla-Kfase	OP700193	Yes	1	1	1	6.8	6.8	6.8	31.111	0	8.7922	248910	1
Moesin-ezrin-radixin homolog 1	L.fla-Moe	OP700194	No	1	1	1	1.7	1.7	1.7	67.572	0.006623	1.4202	232480	2
Protein disulfide isomerase	L.fla-Pdi	OP700195	Yes	2	2	2	5.6	5.6	5.6	55.455	0	2.4932	224740	1
Transmembrane GTPase Marf	L.fla-Marf	OP700196	No	1	1	1	1.4	1.4	1.4	87.555	0	2.2419	208160	2
Isocitrate dehydrogenase	L.fla-IDH	OP700197	No	1	1	1	3.2	3.2	3.2	46.417	0	5.1185	175210	1
Sorbitol dehydrogenase	L.fla-SDH	OP700198	No	1	1	1	4.5	4.5	4.5	38.416	0.006897	1.6295	86907	1
Cadhesin 5	L.fla-Caz5	OP700199	Yes	1	1	1	3.1	3.1	3.1	37.098	0	26.716	0	1
Nucleoside diphosphate kinase	L.fla-NDPK	OP700200	Yes	1	1	1	10.1	10.1	10.1	18.485	0	3.975	0	1
Gamma-interferon-inducible lysosomal thiol reductase-like	L.fla-GILT	OP700201	Yes	1	1	1	16.3	16.3	16.3	9.2604	0	24.587	0	1

Table S3: Comparison of exon numbers, sizes of protein products and presence of domains in *L. lunatus* zonadhesins

	GenBank	Exons	Size (kDa)	Predicted domains (Expasy Prosite)	Comment
L.lun-Zon1	BK062784	17	105	13x EGF_2; 3x ANTISTASIN	2 ANTISTASIN domains overlap with EGF_2
L.lun-Zon2	BK062787	8	49	3x EGF_2	
L.lun-Zon3A	BK062786	4	16	-	
L.lun-Zon3B	BK062809	3	17	-	
L.lun-Zon4	BK062787	10	73	8x EGF_2	
L.lun-Zon5	BK062787	7	38	3x EGF_2	
L.lun-Zon6	BK062793	6	27	1x EGF_2	
L.lun-Zon7	BK062793	5	33	2x EGF_2	
L.lun-Zon8	BK062812	10	54	6x EGF_2	
L.lun-Zon9	BK062805	11	48	2x EGF_2; 4x PACIFASTIN	
L.lun-Zon10	BK062805	13	58	2x EGF_2; 6x PACIFASTIN	
L.lun-Zon11	BK062808	10	53	4x EGF_2; 1x GLYCOSYL_HYDROL_F22_1	
L.lun-Zon12A	BK062787	5	27	4x EGF_2	
L.lun-Zon12B	BK062787	7	40	5x EGF_2	
L.lun-Zon13A	BK062785	10	53	3x EGF_2; 1x GLYCOSYL_HYDROL_F22_1	
L.lun-Zon14	BK062815	7	34	5x EGF_2	
L.lun-Zon15	BK062814	6	30	2x EGF_2	
L.lun-PDCPI	BK062805	7	25	5x PACIFASTIN	

Table S4: List of primers used for qPCR

Gene	Forward primer	Reverse primer
AT24A	TCGCCAGCACCTTCAATACC	GAGGCTCCGGCGATGTTAG
C30A	TGCCCCTTCATCAAAAATCC	CGTCGCAGAAACAACCAGTC
C30C	CGGCTTGGAATTATACAGGTCT	GTTGGCGCTCTTGGCACTA
Caz1	TCTTCCTGGTGTGCCTGATTG	TCTAGTGGTGCATGGTTTCTTCC
Caz2A	CCCAACGACGGACACTATCA	TCTTCGGGGAGCAGCATC
Caz3A	GTGTTCCTGTTCGCCTTTCTG	CCCGCCTAGCTTTGATGC
TSAG18	GTTCCAATACGCTCCGATAATGT	GCTGAAATGGCTGTTGGTAGAA
Caz4	AGGCGCTGGGGTTGTGT	AATTTATCGCCTTTTTGGTGA
Caz5	GCAGGAATCCGCATACG	GTGGTGGGAGTTGTTGTTTGAA
Caz6	CAGGGCGTCAATGCGTATC	GGCTTCGGGTCAGTCACAAT
Caz7	TCTTCTTGGTCGCCTTAGTGG	AGTGGCTGCTGATTTGGTTGA
Caz8	TATTCTTCCTGGTATGCCTCGTG	CCGGTGGTTCCCTTGGTAA
Caz9	AGAAGGGAGGAAATGGAGGTAAC	TGGATGCGCTGCTGGAG
Caz10	CAAAAAGCCTGGAAACAAGAATAA	ATGCCCAAAATGACCGTGAC
Caz11	ATGGCGGAAGCAAAGGAAC	GGTCGGAGTCGTGGATGTGT
Caz12	TGGGGGTGCCTGGTCTG	TTCCCCCGTTGTTGCTATTG
Est1	AAAGTTCGGGAGCGGTGAC	CCCATGGGCATAAACAGGAT
FibH	TCGCAAATCTAACGGAAACTGT	AAAATCGTGATGAGACTGTGAACT
FibL	GACATGGCGCTCTCACTTCTG	GCTCGACTTCGCCTTGTTC
KD15	ATGATGTTGGCTGATTTTATTTAC	TTCGGATTTTATGACTTCTTACAA
LA27	AGGGGCGTACAAAGCAGAGAT	GAGACGCGGGAATTACGAGAT
LAN32	CATGCAACGATTCAAACTACACC	GAACTGCTGCCCAAGACAAGA
PEVK	TCAGCGAGCGTAGAAGACAAGA	CTCACTCGCACCTCCTCCAC
PlipA1	CGTGTCCGCTCTCCTTTCA	AGTTCGTCGTTTTAGTGGCAGTAT
PlipB	ATACTGACGCGTGGGGTTTT	GTTGTATTGCGCGGAAGTGAC
PlipC	CCGTGGCCCAGAAGAAGAT	CCAGAGGCCGATAGTTGAGTGTA
PlipD1	CTTCCGCAGAGCCCAATGT	GCACCGCCTACTAACGAACG
Pxn1	GCCGCCTTCCAGCACAT	GGTAACATCGGGAGCCTTTTT
SMPA1	TCCTCGTGGCCTTTGTGC	ACCGGCTTGTATTTGTGAGACC
SMPA4+5	GGAAAGCCCAAGTATGACAAG	CTATCTATCTACGTTTACCACCTG
SMPA7+8	AACTGACATCATAAACAAGAAAAC	GGATCAAAACTACGTAAGAAATAA
SMPB1	TTTGCTTTATGCTCGCCTCAG	ACTCCCGGATGCCCAATAC
SMPB2	AGTTCCGATTTCTGTTCTTC	CTTTACCCGACCGAGGAG
SP1	ACCGTTGGCCGTTTCCTC	CTGCCCGCCGACTATTCTT
SP2	CTGCGGAGGAACAATCATCAC	AGTTGTTCCGGTAGTTTCAGTTCC
UchC1	GCGATGCCGTTGACAGAAA	TGGCCTAGACCCGCATACTC
YEC1	CTGCAAACCAAGAAAAATCA	CGGGGTCGTAAATGTAGG
YEC3+4	TCCCAGAAATTACGACCCAGTT	TCCTCCCAGCATTCTCCATAGT
Zon1	CCCCAAATGTACCAAGGAAT	GGGGGCAAGCCACATACT
Zon2	CCAAAACCTGCGACAACAA	GTGGAGGTGGGGGACAAT
Zon3B+C	CGGCTCGGAGGACCAGT	TCGGGCCAGACGCAGTT
Zon6	GCCGATTTGATTACTACCGAGATT	TTGGCAACTGGCTTCACCTT
Zon9	TGCGTCACTGGATTCGTATTG	ATTCCGGTGCAGTATTGGTCT
GAPDH	CACCGCCACCCAGAAGAC	GGAGGCGGGGATGATGTT

Supplementary references:

Ashton, N. N. *et al.* Self-Tensioning Aquatic Caddisfly Silk: Ca2+-Dependent Structure, Strength, and Load Cycle Hysteresis. *Biopolymers* **14**, 3668-3681, doi:10.1021/bm401036z (2013).

Frandsen, P. B. *et al.* Exploring the underwater silken architectures of caddisworms: comparative silkomics across two caddisfly suborders. *Philos T R Soc B* **374**, doi:ARTN 2019020610.1098/rstb.2019.0206 (2019).

Luo et al. Exploring the underwater silken architectures of caddisworms: comparative silkomics across two caddisfly suborders. *GigaScience* **7**, 1–12, doi:10.1098/rstb.2019.0206 (2018)

Wang, Y. *et al.* Characterization of unique heavy chain fibroin filaments spun underwater by the caddisfly *Stenopsyche marmorata* (Trichoptera; Stenopsychidae). *Mol Biol Rep* **37**, 2885–2892, doi:10.1007/s11033-009-9847-1 (2010).

Wang, Y. J. *et al.* The silk gland proteome of *Stenopsyche angustata* provides insights into the underwater silk secretion. *Insect Mol Biol*, doi:10.1111/imb.12874 (2023).
